# Setting boundaries for genome-wide heterochromatic DNA deletions through flanking inverted repeats in *Tetrahymena thermophila*

**DOI:** 10.1093/nar/gkz209

**Published:** 2019-03-28

**Authors:** Chih-Yi Gabriela Lin, Ju-Lan Chao, Huai-Kuang Tsai, Douglas Chalker, Meng-Chao Yao

**Affiliations:** 1 Institute of Molecular Biology, Academia Sinica, 11529 Taipei, Taiwan; 2 Genome and Systems Biology Degree Program, National Taiwan University, 10617 Taipei, Taiwan; 3 Institute of Information Science, Academia Sinica, 11529 Taipei, Taiwan; 4 Department of Biology, Washington University in St. Louis, St. Louis, MO 63130, USA

## Abstract

Eukaryotic cells pack their genomic DNA into euchromatin and heterochromatin. Boundaries between these domains have been shown to be set by boundary elements. In *Tetrahymena*, heterochromatin domains are targeted for deletion from the somatic nuclei through a sophisticated programmed DNA rearrangement mechanism, resulting in the elimination of 34% of the germline genome in ∼10,000 dispersed segments. Here we showed that most of these deletions occur consistently with very limited variations in their boundaries among inbred lines. We identified several potential flanking regulatory sequences, each associated with a subset of deletions, using a genome-wide motif finding approach. These flanking sequences are inverted repeats with the copies located at nearly identical distances from the opposite ends of the deleted regions, suggesting potential roles in boundary determination. By removing and testing two such inverted repeats *in vivo*, we found that the ability for boundary maintenance of the associated deletion were lost. Furthermore, we analyzed the deletion boundaries in mutants of a known boundary-determining protein, Lia3p and found that the subset of deletions that are affected by LIA3 knockout contained common features of flanking regulatory sequences. This study suggests a common mechanism for setting deletion boundaries by flanking inverted repeats in *Tetrahymena thermophila*.

## INTRODUCTION

Chromatin structures regulate gene expression, maintenance and transmissions in eukaryotes and are often organized in domains (1). Heterochromatic domains are condensed and silent in transcription with distinctive molecular components. The DNA packaged in these domains can be defined by specific boundary elements, the loss of which leads to spreading of the heterochromatic state into the neighboring region (2). Understanding the molecular nature of domain boundary control is critical to the study of gene activities in chromosomes. In ciliated protozoa, one major form of heterochromatin is believed to govern programmed deletion of thousands of specific DNA segments, thus offering a special setting in which to understand the regulation of chromatin boundaries.

Several *cis*-acting boundary elements have been described in a diverse array of eukaryotes. So-called insulators have been shown to block the propagation of heterochromatin and regulate gene expression (2,3). The propagation of heterochromatin is restricted between the E and I silencers at the silent mating type loci (HML and HMR) in *Saccharomyces cerevisiae*. In fission yeast, there are two inverted repeats flanking the silent region of the mating type loci. Deletions of these elements caused the methylation of histone H3 on lysine 9 (H3K9) to spread into adjacent sequences (4). In the Drosophila 87A7 heat-shock locus, flanking sequences, scs and scs’ (specialized chromatin sequences) contain the binding sites for proteins Zw-5 and BEAF-32, which are responsible for the insulator function (5,6). The highly-conserved protein CTCF (CCCTC-binding factor) has been showed to bind to insulators and block enhancer activities in vertebrates (7–9). These results suggest that the interaction between the *cis*-acting boundary elements and the specific targeting DNA-binding proteins are important in limiting heterochromatin propagation.

*Tetrahymena thermophila* carries out massive DNA deletions that are regulated by chromatin structures. The organism displays nuclear dualism, with a somatic (macro-) and a germline (micro-) nucleus present in the same cell. The macronucleus contains the necessary genetic information for vegetative cell growth and division, and the micronucleus contains all of the inherited genetic materials. During the growth phase, the macronucleus undergoes amitotic division and is actively transcribed while the micronucleus divides by typical mitosis and is transcriptionally silent. During conjugation, the micronucleus goes through mitosis, meiosis, and cross fertilization to generate zygotic nuclei, which further divide and develop into new macro- and micronuclei (10,11). The developing new macronucleus undergoes a series of dramatic programmed DNA rearrangements, including the elimination of ∼34% of the genome (from 157 to 104 Mb) and the fragmentation of the five micronuclear chromosomes into about 225 minichromosomes that are retained in the macronucleus (11,12).

*Tetrahymena* programmed DNA rearrangement was first revealed through comparative genomic DNA hybridization studies (13). Large amounts of sequences were selectively eliminated from the developing new macronucleus, implicating an intricate mechanism of regulation. Two globally occurring processes were later found: IES (internal eliminated sequence) deletion and chromosome breakage, with IES deletion responsible for eliminating the bulk of the germ-line specific sequences. Several lines of evidence have revealed an RNA-guided DNA deletion mechanism that uses small RNAs to guide chromatin modifications to the DNA segments to be targeted for removal (10,11). During conjugation, bidirectional transcripts are generated from selected regions of the micronuclear genome and processed into small RNAs (14–16). These small RNAs target the homologous sequences in the developing macronucleus to trigger histone H3K27 and H3K9 methylation (17,18) and recruit other proteins including Pdd1p, a HP1-like chromodomain protein (18–20). The Pdd1p-containing complex in turn recruits Tpb2p, a domesticated *piggyBac* transposase (21) to execute IES excision (22). The broken ends are rejoined through a nonhomologous end-joining (NHEJ) pathway (23) and result in deletion junctions with certain degrees of sequence microheterogeneity, probably generated from the cutting or the rejoining process (12). Recent studies have discovered a minor pathway that utilize two other domesticated *piggyBac* transposases, TPB1 and TPB6, to eliminate a small subset of IESs that target terminal sequences instead of heterochromatin to carry out precise deletion (24,25).

Since most IES deletions are controlled by heterochromatin, there are probably special domain boundaries to limit the extents of deletions. The nature of this boundary determination mechanism remains largely unknown. It is interesting to note that DNA deletions in *Tetrahymena* can be induced to occur at random locations by dsRNA injection. However, these deletions lack defined boundaries (with variations up to several kbs), and implied the existence of boundary regulatory sequences in natural deletions (26). Indeed, previous studies have reported the existence of flanking regulatory sequences (FRSs) that help determine the boundaries of several IESs. The well-characterized M element has two alternative left boundaries and one shared right boundary (27,28). All three boundaries contain a 10-bp polypurine sequence (5′-AAAAAGGGGG or A_5_G_5_) in their flanking regions a short distance (∼45 bp) away and arranged in opposite orientations, thus appearing as a pair of inverted repeats (IR) for each deletion. Removal of this sequence resulted in the formation of highly variable deletion boundaries, and shifting its location caused the boundary to move with it. These results indicate that the polypurine IR serve as the FRSs of the M element (29,30). Furthermore, recent studies have identified a protein, Lia3p, that recognizes A_5_G_5_ sequences and affects the boundaries of the M-element and 4 other elements that also contained A_5_G_5_ flanking sequences (31). The depletion of LIA3 reduced progeny production after conjugation to 15%, revealing the functional importance of this G-rich sequence binding protein in IES deletions. Detailed analysis has also identified FRSs for the R-element, although their sequence identities have been more complex (32). Moreover, additional analysis has suggested the presence of other FRSs in mse2.9 and Tlr1, which may also involve inverted repeats (33–36).

These cases suggest a possible general mechanism for IES boundary determination in *Tetrahymena* based on *cis*-acting flanking sequences. Using the macronuclear and the micronuclear genome sequence information (12,37), it should be possible to test this idea at the genomic level. Here, we investigated the presence of FRSs for IES deletion using genomic sequences from different inbred strains. We found that the occurrences of deletion were mostly, though not always, conserved among strains and that their boundaries show different degrees of variations. We found specialized sequence structures near IES boundaries that could be linked to boundary determination, and experimentally determined the importance of the most prominent ones. Furthermore, we analyzed the macronuclear genomes of LIA3 mutants and found a large number of IESs that are affected by the mutation, and they appeared to share similar *cis*-acting flanking IRs. This study suggests a general rule for IES elimination in *Tetrahymena* and reveals sequence structures that may mark chromatin domain boundaries.

## MATERIALS AND METHODS

### Cell and cell culture

*Tetrahymena thermophila* inbred strains B2086 II, CU427 (Chx/Chx [VI, cy-s]), and CU428 (Mpr/Mpr [VII, mp-s]) were obtained from Peter Bruns (Cornell University, Ithaca, NY). Homozygous homokaryon Lia3Δ strains (31) were generated by the Chalker lab (Washington University. St. Louis, MO, USA). The method for maintaining and growing cells was as previously described (38). *Tetrahymena* cells were grown in NEFF medium (0.25% proteose peptone [BD, NJ, USA], 0.25% yeast extract [BD], 0.5% dextrose [AMRESCO LLC, OH, USA], 0.022% ferric chloride [Sigma-Aldrich Corp., St. Louis, MO, USA]) at 30°C. Cells were prepared for mating by washing with 10 mM Tris–HCl (pH 7.4) buffer and incubating at 30°C overnight to starve the cell before mixing to initiate mating. After 10 hours of mating, pairs from Lia3Δ strains were picked and transferred individually to drops of SPP for 48 h to allow growth and then replicated to drops with specific drugs to identify progeny cells. Viable progeny cells were transfer to 96 well plates.

### Genomic DNA sequencing and alignment

Genomic DNA was prepared using methods previously described (39). We sequenced the genomes of inbred and Lia3Δ progeny strains to a depth of 49–60 million read-pairs with 2 × 100 bp using Illumina HiSeq 2000 paired-end sequencing (Illumina Inc., San Diego, CA, USA) at the BRC NGS Core Facility in Academia Sinica (Taiwan). Sequencing quality was measured using FastQC software (version 0.11.2; http://www.bioinformatics.babraham.ac.uk/projects/fastqc). Quality scores across all bases were confirmed to be more than 30. Error corrections for reads were using Musket (version 1.0.6) (40). Sequence alignment was mapped into the MIC genome assembly data (Tetrahymena Comparative Sequencing Project BIoHaM, https://www.ncbi.nlm.nih.gov/bioproject/?term=Tetrahymena%20broad%20institute) as the reference genome using BWA (version 0.7.15-r1140) (41), and SAM/BAM file handling was done by SAMtools (version 1.3) (42). The mapped reads were visualized using the Integrative Genomics Viewer (IGV) (43) and analyzed using home-made Perl scripts. The raw sequence data sets have been deposited at NCBI BioProject (http://www.ncbi.nlm.nih.gov/bioproject) as PRJNA326452 and PRJNA416874.

### IES identification

The deletions were first predicted by BreakDancer (44). The distribution of split reads that were extracted from the files of each strain was compared with the predicted deletions. Note that the hard clipping and the soft clipping were both considered, while the average of the clipping counts per position served as the threshold to remove false positives. The position that was near the predicted IES end (within 200-bp window) and had the highest split reads was considered as the reference IES end. Next, the deletions that were less than 100 bp and that contained the unknown nucleotides Ns at the IES ends were removed. The terminal direct repeats, which produce microhomology at each end after cleavage, were moved to the ‘A-end’ of each IES according to their orientation in the MIC genome sequences. The A-end and B-end of an IES refer to the ends that appear in the 5′ and 3′ side of the IES as they appear in the MIC genome sequences.

Two IESs within or among strains that share at least 1-bp overlap were defined as two different forms of the same IES. The boundary variations among IES forms were determined by the sum of the difference at both ends between these two forms.

### Maximum boundary variation

To measure and categorize the variation among different forms of the same IES within and between cell strains, we summed up the length difference at both ends between any two forms. The maximum of these values between any pair of forms for an IES is defined as the maximum boundary variation for this IES. Hence, for an IES, let $diff( {{S_i},{S_j}} )$ be the length difference at both ends between two forms *S_i_* and *S_j_*. The maximum boundary variation of the IES is defined as

\begin{equation*}\mathop {\max }\limits_{{S_i},{S_j} \in forms} diff\left( {{S_i},{S_j}} \right).\end{equation*}

### Flanking regulatory sequence identification

The 100-bp upstream and downstream of IES flanking regions were extracted and the reverse complement of the downstream sequences were used for searching IRs with identical sequences. IRs that were located on both ends and with similar distances to the reference IES ends of CU427 (less than 10-bp difference) were selected, and the occurrences at each position were calculated. The concentricity was defined by IQR (the interquartile range); IQR is represented by the range including the middle 50% of the population, i.e. the difference between the third quartile (75 percentile) and the first quartile (25 percentile). A lower IQR indicated that these IRs were more concentrated in IES flanking regions. The threshold of concentrated pentamer IRs was IQR ≤10 and count ≥3.

### Functional analysis of flanking regulatory sequence

Three DNA fragments of IESs together with 100 bp of flanking sequences on both sides were synthesized by GenScript: the normal sequence and a mutant version without TACCNT from supercontig2.89 (IES: CU427.Supercontig2.89.6054; Supercontig2.89: 310,201–310,615; motif positions and sequences shown in Supplemental Table S2), and the mutant version without C-rich IRs from supercontig 2.504 (IES: CU427.Supercontig2.504.11688; Supercontig2.504: 51,042–51,942; motif positions and sequences shown in Supplemental Table S10). DNA fragment of the normal IES with the same length of flanking sequences from supercontig 2.504 was copied from CU428 genomic DNA by PCR reaction. Supercontig 2.89 and supercontig 2.89 without TACCNT were cloned into the NotI site of the pD5H8 rDNA vector (29). These two insertions were at the opposite direction within the vector. Supercontig 2.504 with or without C-rich IRs were cloned between the PmeI and ApaI site of the pD5H8 rDNA vector.

Biolistic transformation is carried out as previous description (29). Briefly, DNA was coated on 0.6 μm gold particle and delivered to mating cells CU427 and CU428 at 10 hours after mating was initiated using a Biolistic gun (BioRad PSD-1000/He). The transformants were selected by their resistance to paromomycin, and random clones were grown and either pooled or directly examined for their boundary variations using PCR and nucleotide sequencing.

## RESULTS

### IESs are consistently deleted in different inbred strains

**Figure 1.**
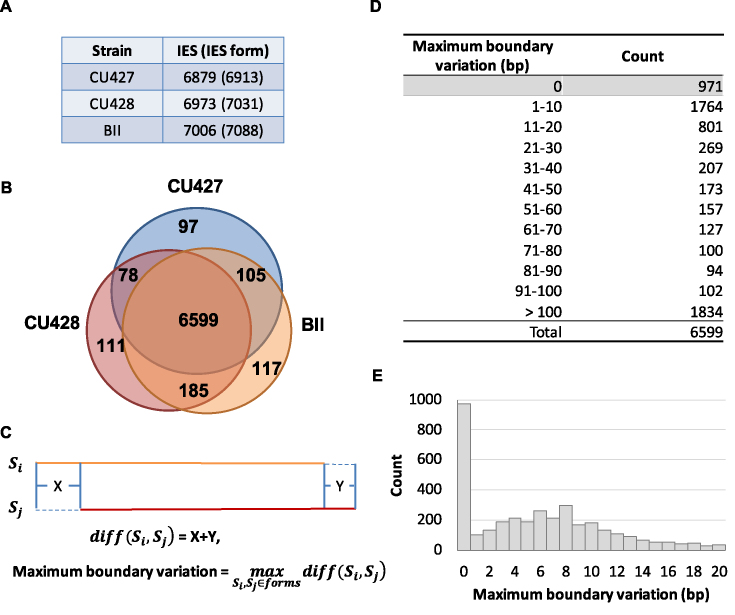
IES elimination among three *Tetrahymena* inbred strains. (**A**) Numbers of IESs and IES forms in the B inbred strains CU427, CU428 and BII. (**B**) The Venn diagram shows that the majority of IESs are shared among the three inbred strains. (**C**) Schematic illustration of the calculations used for IES variations at one location. S: form; X and Y: the boundary variation on each side. (**D**) Boundary variation classes of IESs. Note that intra-strain variations at the same location are included. (**E**) The histogram shows the distribution of IESs with boundary variation within 20 bp.

In order to understand IES boundary determination, we need to first compare the deletion of IESs among different *Tetrahymena* strains to determine their variations. The MAC genomes of three inbred B strains, CU427, CU428 and B2086 II (BII), were sequenced using Illumina paired-end sequencing. To locate IESs that were deleted, we mapped reads onto the MIC reference genome and used BreakDancer (44), a tool for predicting genomic structure variation, to detect deletions from the MIC genome in each MAC genome (12,45). There were 10,127, 10,176 and 10,138 deletions detected in CU427, CU428 and BII, respectively. We observed that the deletion boundaries predicted by BreakDancer did not offer sufficient precision, hence, we improved the resolution by extracting split reads located at each junction and used them to identify the exact nucleotide position of the breakage point. Many deletions contained unknown nucleotides at the junction due to incomplete micronuclear genome sequences and were removed. After these refinements, 6913, 7031 and 7088 deleted segments were identified with high confidence in CU427, CU428 and BII, respectively (Figure 1A). During this process, we observed that some deleted segments shared significant overlaps and should be considered alternative forms of the same IES, indicating that a small population of IESs have intra-strain variation. They were further verified by identifying the mapped reads across the junctions. Hence, the number of non-overlapping IESs identified were 6879, 6073, 7006 in CU427, CU428 and BII, respectively (Figure 1A), including some well-defined IESs that are TPB1-dependent (Supplemental Table S1).

Next, we compared the occurrence of deletions among these strains, and found that the deletion of >95% of IESs (averaging 6733 IESs) are shared by any pair of strains (Supplemental Figure S1) and that more than 94% of IESs (6599 IESs) are deleted in all strains (Figure 1B). This result indicates that the vast majority, but not all, of IESs are consistently deleted in independently developed macronuclei. We further analyzed the publicly available genome data of another B strain, SB210, and found a similar result (Supplemental Figure S2) (12).

Interestingly, there were 85 IESs that had, within a single strain, more than one form of deletion, and for one IES up to nine forms were found (Supplemental Figure S3A and B). Furthermore, for four of these IESs more than one form was found in all three strains, indicating the persistence of multiple rearranged forms at these loci. Note that the developing macronucleus has endoduplicated to a level of about 4–8C when IES elimination occurs, allowing up to eight different deletion forms to be generated at each IES location. Presumably different forms (like different alleles) should be sorted out through amitosis during macronuclear division. These inbred strains have been propagated asexually for many decades, and thus have ample opportunities for assortment. The retention of multiple forms including some that overlapped with expressed genes, especially in all strains, raised the possibility of functional roles for these boundary variations (Supplemental Figure S3C).

### The majority of deletion boundaries show inter-strain microheterogeneity

Since the great majority of IESs were deleted in all inbred strains tested, we next examined their junction sequences for possible inter-strain variations. For each IES, the combined difference in length at both ends between any two strains was calculated and the maximum value was used to indicate the extent of its boundary variation (Figure 1C). For instance, if there were three forms for an IES and the junction difference were 30, 40 and 50 bp between each pair of forms, this IES was put into the group with 41-to-50-bp variation. The results show that the junctions of deletion varied from 0 to 56,391 bp, with 14.71% of IESs showing no boundary variation (Figure 1D and E), 38.87% exhibiting variations of 1-to-20-bp, and 27.79% differing by more than 100-bp (Figure 1D). This result indicates that the majority (53.58%) of IESs showed very limited boundary variations during deletion (20 bp or less).

### Abrupt change in nucleotide distributions near IES boundaries suggests potential *cis*-regulatory sequences

**Figure 2.**
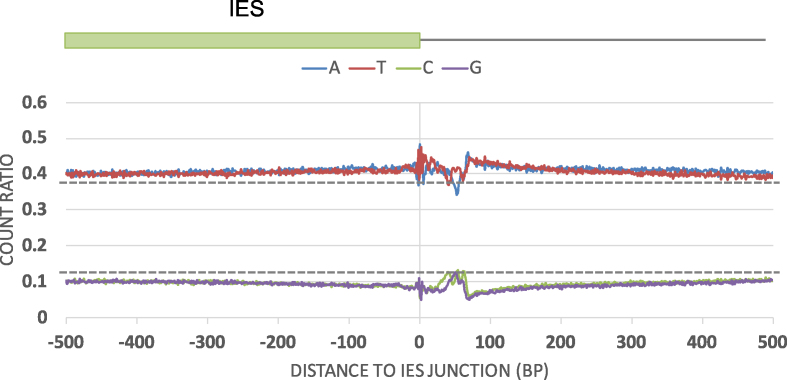
Nucleotide distribution near IES boundaries. The plot shows the nucleotide distribution of the first 500-bp sequences of all IESs next to an end and the adjacent 500-bp flanking sequences in CU427. Sequences surrounding both ends of all IESs were used in the compilation. Zero indicates the boundary of IESs. The upper and lower dashed lines indicate the average genomic contents of A or T and G or C, respectively.

To explore the possibility that *cis*-regulatory sequences are commonly used to determine IES boundary, we searched for nucleotide sequence patterns near mapped junctions. We first aligned all 6599 IESs according to their deletion boundaries and examined the nucleotide distribution at each position within 500-bp on each side of the reference end of CU427 (within the IES and in the flanking region). These regions contained a slightly lower GC content than the MIC genome average (25%GC), presumably due to the largely non-coding nature of IESs and their immediate flanking regions. Strikingly, abrupt and significant changes were observed for a short (∼50 bp) interval within the first 100-bp of the flanking regions. This interval includes the locations in which the flanking polypurine sequences of the M-element were located. This result strongly suggests that boundaries of a significant proportion of IESs are marked by special flanking sequences (Figure 2), which have the potential to play a regulatory role.

### Inverted Repeats near IES boundaries as potential regulatory sequences

**Figure 3.**
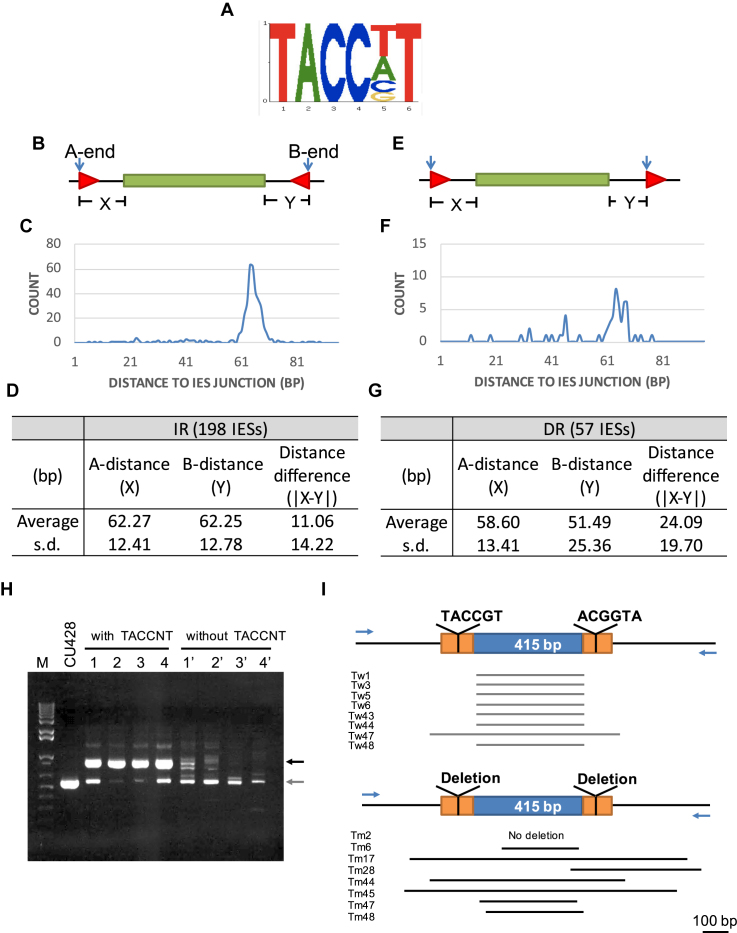
IR of the motif ‘TACCNT’ at similar distance to both ends of IESs. (**A**) Conserved sequence of ‘TACCNT’. (**B**) A cartoon shows the arrangement of IR that flanks an IES. (**C**) Tight distance distribution of the motifs as IRs near IESs in the CU427 genome. (**D**) Statistic information of the ‘TACCNT’ IRs in CU427. (**E**) A cartoon shows the arrangement of DR. (**F**) Distance distribution of the motifs as DRs near IESs in CU427. (**G**) Statistic information of the ‘TACCNT’ DRs in CU427. A-distance: distance of motif to one end of the IES; B-distance: distance of motif to the other end of the IES; distance difference: difference of the distances of the motif to either end of the IES; s.d.: standard deviation. (**H**) PCR of genomic DNA isolated from clones of IESs with or without the flanking T-domain. Dark arrow: expect arranged form; gray arrow: unspecific band. (**I**) Diagram of IES regions based on the sequencing result. Blue arrow: position of the primer set. Tw: single clone of WT IES with T-domain; Tm: single clone of mutated IES without T-domain. Arrow: primer.

To identify potential ‘flanking regulatory sequences’ that may help set the boundary, we searched for shared sequences with particular features, using motif finding tools, eTFBS and MEME (46,47). For eTFBS, flanking regions within 100-bp from IES ends in CU427 were scanned to find 10 overrepresented motifs that contained the longest conserved sequences (Supplemental Figure S4A). The IES flanking regions between 100 and 200 bp away from the junctions were used as the background dataset. Most of the motifs identified had high AT patterns, supporting the higher AT content of the 100-bp flanking regions to the background. However, most of them did not display other common features, except two (motifs 2 and 7) that displayed a consistent distance to reference IES ends when occur as IR but not as direct repeats (DR). These two motifs share the same core sequence ‘TACCNT’ (Figure 3A). Coincidently, the ‘TACCNT’ motif (Top 7) was also predicted as a significant motif by MEME using the sequences within 100-bp flanking regions of the IESs in CU427 (Supplemental Figure S4B). There were a total of 1881 copies of these motifs in the 100-bp flanking regions of all IESs, of which 198 occurred at both sides of an IES as IR and 57 as DR (Figure 3D and G and Supplemental Table S2). Significantly, these IRs occurred at similar distances (∼62 bp) to reference IES ends, with an 11 bp variation on average between the two sides of the same IES (Figure 3B and C). This common pattern was not found for the DRs (Figure 3E and F).

To directly test whether the TACCNT motif acts as a FRS *in vivo*, we adopted an assay routinely used to examine the *cis*-requirement for IES excision and inserted an IES flanked by the TACCGT IR (referred to as T-domain for the following TACCNT IR) into an artificial rDNA mini chromosome transformation vector (29). After introduction of these vectors into *Tetrahymena* cells during conjugation, any deletion that occurred in this construct can be detected in the transformed progeny. IES constructs with or without the flanking T-domain were generated and tested and their deletion boundaries determined using PCR and nucleotide sequencing. As expected, the normal IES with the T-domain showed highly regulated boundaries in the clones analyzed. Consistent with the hypothesis that this sequence controls the accuracy of excision, the mutated IES lacking the T-domain lost it defined boundary as excision became highly variable (Figure 3H, I and Supplemental Table S3). This result indicates that the TACCNT motif is an essential FRS that controls the boundary of this and likely other IESs with a similar flanking sequence motif.

We noticed interesting common features between the T-domain and the polypurine sequences of the M element: they are both IRs at similar distances to respective reference IES ends. We thus repeated the search by focusing on IRs that were located at similar distances (less than 10-bp difference) from the two ends of an IES in the CU427 genome dataset. We arbitrarily defined an IR as a pair of pentamer sequences with no mismatches between the copies flanking each IES. We clustered these IRs and determined the distributions of their left copies relative to their proximal IES ends. Since the locations of two copies were similar to the reference IES ends, we assumed that the location distribution of the copy on the right-hand side was similar with the left-hand side at this step. We identified 472 pentamer sequences that occurred as IRs at the flanking regions. The pentamer ‘ATTTT’ IR occurred at the highest frequency; however, it was widely dispersed with no apparent pattern (Supplemental Figure S5). On the other hand, we found 136 pentameric IRs with their distributions concentrated within a small range (Supplemental Table S4).

Interestingly, when some groups with high concentrated distributions that shared the same core sequence were combined, their concentricity was still maintained. There were 2700 IESs that contain pentamer IRs with the core sequence ‘TATA’, which was the most frequent group with high concentrated distribution (Supplemental Figure S6A and Supplemental Table S4). These pentamer IRs had a tight distribution that were about 65-bp away from the reference IES ends (Supplemental Figure S6B). Other cases also showed the same property of having the IR at similar distances from both ends of the IESs (Supplemental Figure S6C-F), implying a relevant relationship between the location of the IRs and the IES boundaries. In addition, the above identified T-domain were also grouped as high concentrated IRs where both the ‘TAC’ and the ‘TACC’ groups include the ‘TACCNT’ IRs (Supplemental Figure S7A–C), indicating that this method can sufficiently identify the consensus of IRs that showed distinct patterns near the IES flanking regions. Moreover, pentamer IRs composed of G or C were also highly represented in the high concentrated groups (Supplemental Figure S7D-F). The common feature of the concentricity of the distance of IRs to the IES boundaries within the same groups suggests that these IRs may represent a type of FRSs for IES boundary determination.

Altogether, 3794 IESs were included in the 6 major IR groups mentioned above, which covered 57.49% of all IESs shared among the three inbred strains. This result implied that IRs could be the major type of regulatory sequences for IES boundary determination.

### Lia3p regulates a distinct subset of IESs

**Figure 4.**
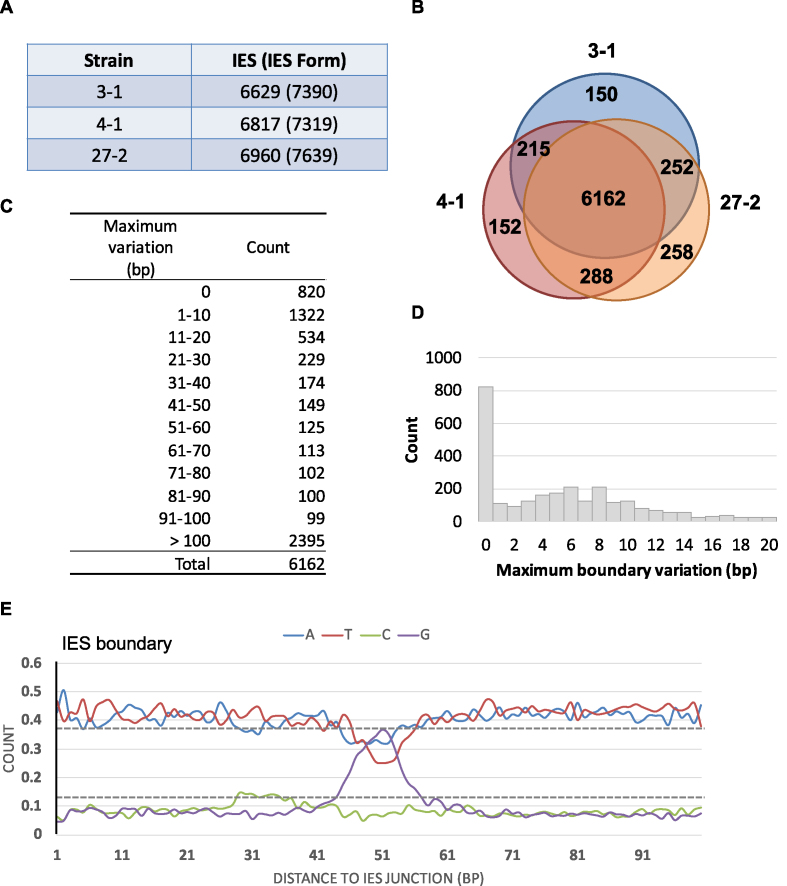
IES elimination among Lia3Δ strains. (**A**) Numbers of IESs and IES forms in Lia3Δ strains 3–1, 4–1 and 27–2. (**B**) Venn diagram showing that the majority of IESs are shared among the three Lia3Δ strains. (**C**) Boundary variation classes of IESs in the LIA3Δ strains. Note that intra-stain variations at the same location are included. (**D**) IES Boundary variation classes within 20 bp in LIA3Δ strains. (**E**) A plot shows the nucleotide distribution of flanking sequences near both ends of 387 Lia3-affected IESs in CU427. To generate this figure, we used a stringent definition of Lia3-affected IESs, i.e. those having ≤100-bp variation among the three inbred strains and increase by >100-bp variation among the three LIA3Δ strains. The upper and lower dashed lines indicate the average content of A or T and G or C, respectively.

Lia3p was recently shown to control the position of boundaries of the M element by binding to its G-rich FRS (31). LIA3-deficient cells also exhibited imprecise deletion boundaries for five other IESs that had similar G-rich sequences as the M element. We suspected that Lia3p may control many more IESs, many of which could include the IESs we found with G-rich IRs (Supplemental Figure S7D). To reveal the spectrum of IESs with boundaries controlled by Lia3, we generated three progeny lines (3-1, 4-1 and 27-2) from the LIA3Δ strains and sequenced their macronuclear genomes to identify defects in IES boundaries (Figure 4A–D). Since these LIA3Δ strains were also derived from the B inbred lines, we used the three B inbred lines described earlier for comparison (Supplemental Figure S8). We noticed that the total number of IESs with >100-bp boundary variations was 11.08% higher in these mutant strains (38.87%) than in the inbred strains (27.79%) (Figure 4C), suggesting that Lia3p may regulate a large number of IESs.

### G and C are enriched near the boundary of LIA3-affected IESs

To identify the subset of IESs with boundaries controlled by Lia3p, we compared IES variations among inbred strains and LIA3Δ progeny lines, which revealed 519 IESs that showed higher (by at least 100-bp) boundary variations in these LIA3Δ progeny lines (Table 1). They are referred to as ‘LIA3-affected IESs’ thereafter. To look for possible common motifs, we extracted the 100-bp flanking regions from both sides of IESs in this group and calculated the nucleotide ratios in each position. To reduce potential noises caused by IESs that were highly variable even in the inbred strains, we only considered a subset (387 of the 519 IESs) that showed at most 100-bp variation among the inbred strains (Supplemental Table S5). Remarkably, we found a distinct enrichment of G at positions 40–60 bp away from the reference IES ends of CU427 (Figure 4E). It agrees very well with the characteristics of FRS of the M-element, and further supports their potential role in the regulation of deletion boundaries. Unexpectedly, we also observed a small peak of Cs at positions 25–40 bp from the reference IES ends (Figure 4E), suggesting the possible existence of some C-rich motifs under Lia3p regulation.

**Table 1.** Number of IESs exhibiting ≥100-bp boundary variations between the three inbred and three Lia3Δ strains

Maximum variation (bp)	Total IES*	LIA3-affected IES	Ratio
**0**	816	86	0.11
**1–10**	1486	144	0.10
**11–20**	659	78	0.12
**21–30**	231	18	0.08
**31–40**	169	13	0.08
**41–50**	146	11	0.08
**51–60**	128	11	0.09
**61–70**	103	13	0.13
**71–80**	75	6	0.08
**81–90**	75	2	0.03
**91–100**	82	5	0.06
**>100**	1393	132	0.09
**Total**	**5363**	**519**	**0.10**

*In each variation category, total IESs include the LIA3-affected IESs. Only IESs that are shared by the three WT strains and the three LIA3Δ strains are included in this tabulation.

### G-rich and C-rich inverted repeats at the flanking regions of LIA3-affected IESs

**Figure 5.**
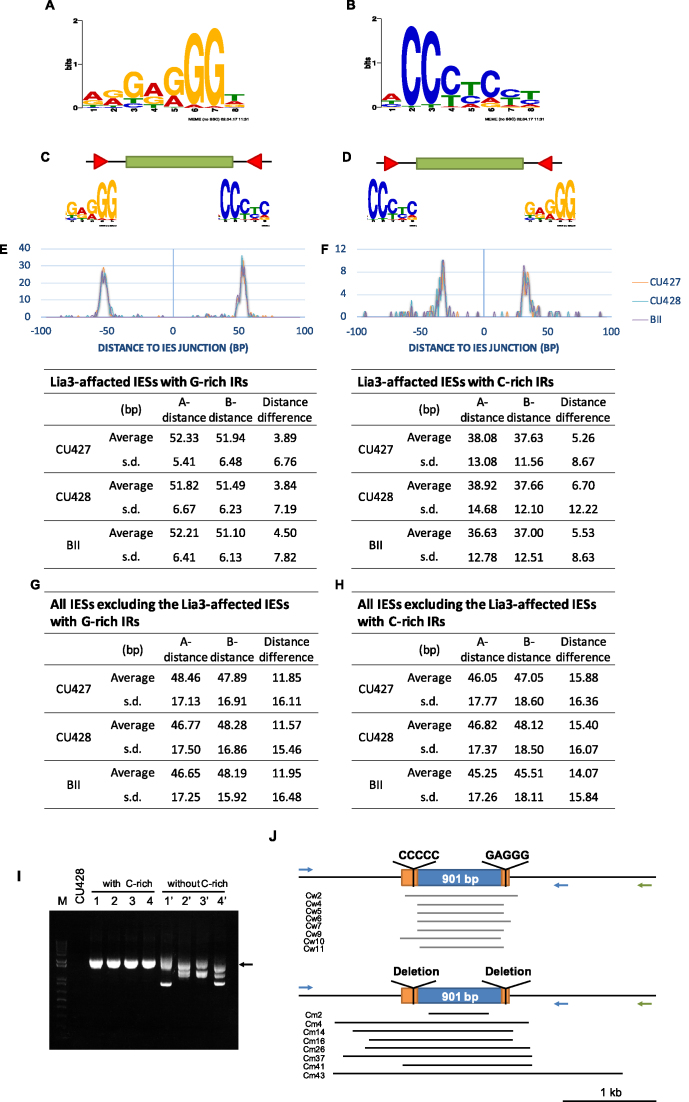
G-rich IR and C-rich IR are positioned as the flanking regulatory elements in LIA3-affected IESs. (**A** and **B**) Shared sequence motifs (analyzed by MEME) in 308 LIA3-affected IESs that show very limited variation (20-bp or lower) among the inbred strains. LIA3Δ strains increased the variations by at least 100-bp. (B) represents the reverse complement of the motif in (A). (**C** and **D**) Cartoons show the arrangement of G-rich or C-rich IR, respectively. (**E** and **F**) Motifs of the G-rich IR and C-rich IR in LIA3-affected IESs that increased the variations by at least 100-bp of IESs that show lower degrees of variation among inbred strains. We defined 75% similarity as the minimum score of the PWM indicated in (A) for the threshold. (**G** and **H**) Motifs of the G-rich IR and C-rich IR from the entire IES dataset, respectively. The numbers of LIA3-affected IESs indicate in (E) and (F) have been removed. A-distance: distance of the motif to one end of the IES; B-distance: distance of the motif to the other end of the IES; s.d.: standard deviation. (**I**) PCR of genomic DNA isolated from clones of IESs with or without the flanking C-rich IRs. Dark arrow: the expected rearranged form for normal deletion. (**J**) Diagram of IES regions based on the sequencing result. Blue arrow: position of the primer set. Cw: single clone of WT IES with C-rich IRs; Cm: single clone of mutated IES without C-rich IRs. Arrow: primer. Noted that the proximal (blue) and the distal (green) reversed primers in the right flanking region were individually paired with the forward primer in the left flanking region in separate PCR tests.

We then searched for common motifs within these 100-bp flanking regions using MEME (47), and identified conserved G-rich sequences (Figure 5A). To minimize background noise we only used the subset of 308 LIA3-affected IESs that showed very low variation (at most 20 bp) among the inbred strains. Note that the conserved sequences predicted from MEME combined both orientations of the motif (both G-rich and C-rich sequences). We then determined the enrichment of these G-rich or C-rich motifs in the 387 IESs that were affected by LIA3. As summarized in Figure 5, there appears to be a strong correlation between LIA3 effects and the presences of G-rich or C-rich IRs with high concentricity, strengthening the possibility that Lia3p acts through these IRs.

To refine the IR sequences associated with LIA3 effects, we tested different similarities of PWM (position weight matrix) value from the consensus we built in Figure 5A and B in CU427 and found that in the G- or C-rich IRs with 75% similarity, >60% of Lia3-affected IESs contained one of the two IRs, but only ∼14% of the background IESs contained them (Supplemental Table S6). This is a very robust correlation. We thus set the PWM threshold at 75% similarity for subsequent experiments. It should be noted that if we lowered the threshold to 60% similarity, 91.21% of Lia3-affected IESs were found to contain G-rich or C-rich IR (Supplemental Tables S6 and S7). However, this would also increase the background to 73%, reducing the distinction between these two groups.

Next, we scanned the flanking regions of these 387 LIA3-affected IESs for the two IRs. We analyzed all three B strains, and the results were quite similar. Significantly, 59.95% of Lia3-affected IESs had G- or C-rich IRs among all inbred strains, but only 11.29% of the background IESs had these IRs (Table 2 and Supplemental Tables S10 and S11), indicating a strong correlation between these IRs and LIA3 effects. It is noted that if the G- and C-rich IRs appeared in the same IES, the one with lesser distance differences between both ends of the IES was assigned as the FRS of the IES. However, the overlaps were rare. In CU427, only seven LIA3-affected IESs appeared to have the two IRs at both ends. Moreover, the distances between these IRs and the reference IES ends were very similar among IESs (Figure 5E and F), and especially between the two ends of the same IESs (*P*-value<10^−5^ on average). This consistency was absent from those IESs unaffected by LIA3 (but have G- or C-rich IRs) (Figure 5G and H).

**Table 2.** Numbers of LIA3-affected IESs in all three B strains

IES no.: 387	Similarity^b^: 75%		LIA3-affected candidate IESs				LIA3-unaffected IESs		
Constrain^a^	Strain	G IR^c^	C IR^d^	Total	%	G IR^c^	C IR^d^	Total	%^e^
WT ≤ 100 (WT-Lia3Δ) ≥ 100 bp	CU427	175	62	237	61.24	295	400	695	14.02
	CU428	175	61	236	60.26	252	395	647	13.00
	BII	175	62	237	60.26	283	465	748	15.03
	At least one strain^f^	177	63	240	62.02	376	566	942	18.93
	All strains^g^	171	61	232	59.95	186	376	562	11.29

^a^Difference between IESs among three inbred strains (WT) is less than or equal to the indicated number of base pairs (bp), and the IES differences between the three WT and three Lia3Δ strains are ≥100 bp.

^b^Similarity of PWM score of the consensus indicated in Figure 5A and B.

^c^Number of IES candidates containing G-rich IRs.

^d^Number of IES candidates containing C-rich IRs.

^e^Percentage of IESs in the WT background without candidates of G-rich and C-rich IRs.

^f^Values indicate that at least one strain contains the IES with the indicated IR.

^g^Values indicate that all three strains contain the IES with the indicated IR.

We also considered the G-rich or C-rich motifs as DRs. We identified 41 G-rich DRs and 45 C-rich DRs in the group of LIA3-affected IESs. However, their distances to the reference IES ends were less consistent (53.32 bp ± 23.24 in G-rich DRs and 50.12 bp ± 22.54 in C-rich DRs). In addition, the two copies flanking the two ends of an individual IES showed higher distance variation for DRs (∼25 bp in G-rich DRs and about 17 bp in C-rich DRs) than for IRs, making DRs less likely to serve as boundary regulatory elements. Our results show that LIA3-affected IESs are likely regulated by the IRs of G-rich or C-rich sequences.

To determine if the predicted C-rich motif is indeed a FRS, we generated constructs of an IES with the C-rich IR and its mutant without the IR, and examined their deletion boundary maintenance in *vivo*. The results show that the boundaries become highly variable in the mutant lacking the C-rich IRs (Figure 5I and J). In conclusion, we found that not only the G-rich IR, but also the C-rich IRs function as FRSs in LIA3-affected IESs.

### Multiple flanking regulatory sequences exhibited in LIA3-affected IESs with G-rich and C-rich IRs

Some IESs that are likely controlled by Lia3p actually show large boundary variations even in normal strains. This could indicate that some IESs have relaxed boundary control or, alternatively, some IESs may exhibit precisely controlled alternative boundaries. This scenario has been described for the M element, which can undergo two equally likely deletion outcomes, removing either 0.6- and 0.9-kb (48). The two forms have the same right boundary but different left boundaries that are 0.3-kb apart, and each boundary contain the 5′-A_5_G_5_ motif positioned ∼45 bp away (30). Consistent with the possibility that junction variability in wild-type cells represents control of alternative junctions, closer examination revealed that variable junctions each had copies of the same putative FRSs. We observed that a potential FRS could usually be found at a consistent distance to an IES boundary even if the boundary variation was high, suggesting that the same tight distance control was maintained. Supplemental Figure S9 showed another example of a LIA3-affected IES that contained one copy of the G-rich IRs near the right junction and two copies near the left junctions. The two forms of deletion in CU427 and BII used the same outer pair of IR and generated deletions with only 1-bp variation at the right junction, while the single form in CU428 used the inner pair of the IR and showed 61-bp difference at the left junction and 1-bp difference at the right junction from the other two strains. A simple survey revealed 40 and 8 IESs with multiple FRSs in the LIA3-affected IESs with G- and C-rich IRs, respectively, representing 72.73% and 88.9% of the respective group with more than 20-bp variation (Supplemental Table S8). Moreover, a T-domain containing IES with the highest level of variation (Table S2, CU427.Supercontig2.222.9221) was actually deleted as two segments that were 908-bp apart in CU428 and BII but as one continuous form in CU427 (Supplemental Figure S3A and Table S9). T-domains or its degenerated sequences were found in most of the flanking regions of these 3 IES forms, suggesting that alternative deletions can occur when several combinations of FRSs are available in the same region.

We described in an earlier section that nearly half of the IESs showed >20-bp variation. The alternative deletion just described may offer a potential explanation. Looking at the genome-wide situation, we were surprised to find that 5573 of these 6599 IESs (84.45%) showed little or no variations (≤20-bp variation) in at least one end. It is likely that there are limited numbers of defined potential boundaries for most IESs. This result implies that the majority of IES boundaries are well regulated and those that do vary may partly be due to the alternative use of multiple FRSs that are present in these IESs. Among the 380 LIA3-affected IESs, about 73% contained the alternative deletions in at least one end of the new boundary when LIA3 was mutated (Supplemental Table S5), raising the possibility that secondary FRSs and their interacting proteins are used to set boundaries when Lia3p is depleted. Altogether, our finding supports the mechanism that IES boundaries are determined by flanking regulatory sequences.

## DISCUSSION

In this study we investigated the global regulatory mechanism of IES deletion boundary determination. We observed that the occurrence of deletions was highly, though not completely, conserved among different *Tetrahymena* strains. For those conserved IESs, the majority of deletion boundaries exhibited microheterogeneity of 20 bp or fewer at each end. In searching for potential regulatory sequences we discovered that each of several IRs is present outside a subset of IESs, with each copy of the two repeats located at nearly equal distances from each end of an IES. These two copies likely work as a pair. Earlier studies that manipulated these sequences of the M-element have also suggested this possibility (29,30). This finding suggests that the boundaries of these IESs can be determined by a mechanism with these IRs serving as flanking regulatory sequences. Thus, the majority of *Tetrahymena* IESs, which are specified by heterochromatin, could have their boundaries determined by flanking regulatory sequences to limit their variations.

Although the vast majority of IESs are deleted in all strains analyzed, there are 693 IESs that are deleted only in one or two strains. This interesting variation could be caused by at least two possibilities. Firstly, the execution of deletion could be inefficient and only some of the copies in the polyploid MAC are deleted. Random assortment of these copies during cell growth and amitosis could generate clones with or without the deletion. Secondly, the interesting epigenetic effects exerted by the parental MAC could inhibit deletion in some strains (17). There is also a technical issue to consider that is related to the detection of IESs by BreakDancer. It could be less consistent in particular regions of the genome and contributed to this variation. We have randomly selected ∼10% of IESs from this group for analysis by individual inspection and can unambiguously verify ∼30% of them were true positives. It will be interesting to find out how these events are generated and whether this somatic diversification affects cellular fitness.

**Figure 6.**
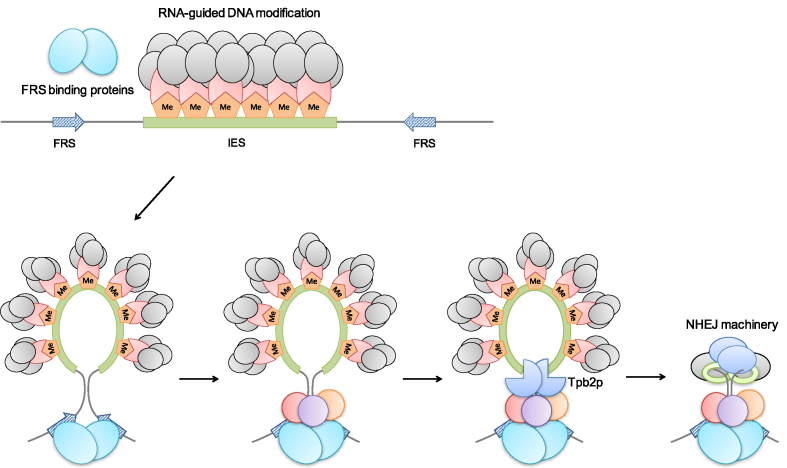
A speculation on boundary regulation of IES deletion. We hypothesize that once the heterochromatin is formed through the small RNA mediated process, its boundaries are set by the interaction between the FRS binding protein such as Lia3p (light blue ovals) and the FRSs (arrow). Together they recruit other proteins (pink, purple and orange circle) including Tpb2p that carries out DNA cutting and IES excision. After IES deletion, the FRS binding protein may also protect the macronuclear-destined region and maintain the two broken ends in close proximity to facilitate the NHEJ process. Without FRSs, the heterochromatin boundaries may spread out, and Tpb2p will cut at variable point to excise IESs (26). Furthermore, the broken ends are not well protected and are eroded before rejoining, causing additional boundary variation.

The nearly identical distances of the two copies of an IR to the IES ends raised the possibility that these two copies could cooperate with each other. We speculate that IRs interact with their binding proteins to set chromatin domain boundaries, which then recruit Tpb2p to cut at these ends. After Tpb2p directed excision, this structure could further protect the macronuclear-destined regions from nuclease digestion and maintain these two double-stranded ends in close proximity before they are joined by NHEJ (Figure 6). Since new boundaries that are formed after the removal of the T-domain, the C-Rich IR, or the depletion of Lia3p are not at entirely random locations but tend to be clustered, we favor the possibility that secondary or alternative FRSs are used once the predominant FRS becomes non-functional, so as to reduce the risk of spreading deletions to nearby coding regions.

Lia3p was the first known example of regulatory proteins to interact with FRSs, and provides an excellent stage from which to further analyze this process. Our genomic analysis of LIA3 mutants revealed a large group of potential IES targets. Surprisingly, we observed that there are two FRSs in LIA3-affected IESs, namely G-rich and C-rich IRs. More than 90% of the LIA3-affected IESs contained at least one of the FRSs under our threshold of 60% similarity of the PWM score, whereas about 60% of them contained one of the FRSs under a threshold of 75% similarity. This finding indicates that almost all of the 387 LIA3-affected IESs contained G-rich or C-rich IRs, though some exhibited lower similarity. Interestingly, the distances between the IRs and the IES boundaries differed between G-rich and C-rich IRs (51 and 38 bp, respectively). A recent study showed that Lia3p preferentially binds to single-stranded sequences with five guanine residues, which forms a parallel G-quadruplex *in vitro* (31), but its ability to bind C-rich sequences is very poor, suggesting that Lia3p bind to the G strand in both G-rich and C-rich IRs. Interestingly, the represented motif in our study was ‘GAGGG’, which has been shown to have the most unstable form for maintaining the G-quadruplex structure (49), suggesting potential structural differences from the conventional G-quadruplex. We suspect that the different orientation of the G strand toward the IESs between these two IR types may affect Lia3p dimerization and alter the distances from the TPB2 cutting site.

Our results suggest that *Tetrahymena* has evolved a special way to harness transposases for IES eliminations. The domesticated *piggyBac* transposases TPB1/6 are responsible for the excision of 12 special IESs with features of transposons (such as terminal inverted repeats [TIR] and the TTAA cutting site) (24). Tpb2p, on the other hand, has lost its ability to recognize the TIR and has evolved to broaden its target sites by recognizing heterochromatin to cut at its boundaries. Here we revealed many IRs in the genome that could serve as potential FRSs to define the excision boundaries. We speculate that FRSs and their binding proteins may have evolved from existing regulatory components of DNA activities, such as transcription factors and their binding sequences or other transposable elements. They are adopted by the IES elimination machinery once a new heterochromatic region arises from genetic agents that has invaded the genome.

To support the idea derived from sequence analysis, we directly tested two of the newly identified FRSs, TACCNT and C-rich motif, and clearly demonstrated their functions to set boundaries *in vivo*. Interestingly, when the flanking ‘TACCNT’ IR was removed and the boundary became highly variable, we noticed that there were FRS-like IRs adjacent to the newly formed boundaries (Supplemental Table S3), suggesting that a similar mechanism is involved in setting a new boundary. Our search discovered that about 60% of IESs contained one of the six main groups of IRs in their flanking regions. By using this system, we argue that TPB2-dependent IES elimination could regulate >6000 IESs by targeting heterochromatin whilst also maintain high degrees of boundary precision.

For the IESs that do not belong to the six groups, we assumed that different kinds of sequence structures might be present in the flanking regions of IESs but were hard to detect through sequence analysis (e.g. the R element). In addition, in this first genome-wide search, we choose to use a stringent method for the motif discovery process, which did not cover all IESs but only the highly confident ones. Our study provides a comprehensive understanding of the IR-regulated IES boundary determination. Further studies will hopefully reveal its detailed mechanism.

This study further supports a remarkable similarity between the mechanism of programmed DNA rearrangements of *Tetrahymena* and that of the V(D)J recombination of the vertebrate adaptive immune system (50). They both target inverted repeats and use domesticated transposases to perform excisions, and repair the break by the NHEJ pathway. The variable combination among the VDJ region resembles the alternative deletion of the IES region. Importantly, as a consequence every *Tetrahymena* cell has a different genome sequence with potentials to adapt to environmental changes, much like the adaptive immunity generated by B and T cells. This convergent evolution is interesting and could advance our understanding of vertebrate immune system and *Tetrahymena* biology.

## DATA AVAILABILITY

The raw sequence data sets have been deposited at NCBI BioProject (http://www.ncbi.nlm.nih.gov/bioproject) as PRJNA326452 and PRJNA416874.

## Supplementary Material

gkz209_Supplemental_Files

## Notes

Present address: Chih-Yi Gabriela Lin, Swiss Institute for Experimental Cancer Research, School of Life Sciences, Ecole Polytechnique Fédérale de Lausanne (EPFL), 1015 Lausanne, Switzerland.

## References

[1] Dillon N. Heterochromatin structure and function. Biol. Cell. 2004; 96:631–637. 15519697 10.1016/j.biolcel.2004.06.003

[2] Bell A.C., West A.G., Felsenfeld G. Insulators and boundaries: versatile regulatory elements in the eukaryotic genome. Science. 2001; 291:447–450. 11228144 10.1126/science.291.5503.447

[3] Donze D., Kamakaka R.T. Braking the silence: How heterochromatic gene repression is stopped in its tracks. Bioessays. 2002; 24:344–349. 11948620 10.1002/bies.10072

[4] Noma K.-I. Transitions in distinct histone H3 methylation patterns at the heterochromatin domain boundaries. Science. 2001; 293:1150–1155. 11498594 10.1126/science.1064150

[5] Cuvier O., Hart C.M., Laemmli U.K. Identification of a class of chromatin boundary elements. Mol. Cell. Biol. 1998; 18:7478–7486. 9819433 10.1128/mcb.18.12.7478 PMC109328

[6] Zhao K., Hart C.M., Laemmli U.K. Visualization of chromosomal domains with boundary element-associated factor BEAF-32. Cell. 1995; 81:879–889. 7781065 10.1016/0092-8674(95)90008-x

[7] Bell A.C., West A.G., Felsenfeld G. The protein CTCF is required for the enhancer blocking activity of vertebrate insulators. Cell. 1999; 98:387–396. 10458613 10.1016/s0092-8674(00)81967-4

[8] Ohlsson R., Renkawitz R., Lobanenkov V. CTCF is a uniquely versatile transcription regulator linked to epigenetics and disease. Trends Genet. TIG. 2001; 17:520–527. 11525835 10.1016/s0168-9525(01)02366-6

[9] Saitoh N., Bell A.C., Recillas-Targa F., West A.G., Simpson M., Pikaart M., Felsenfeld G. Structural and functional conservation at the boundaries of the chicken β-globin domain. EMBO J. 2000; 19:2315–2322. 10811622 10.1093/emboj/19.10.2315 PMC384375

[10] Yao M.-C., Chao J.-L. RNA-Guided DNA deletion in tetrahymena: an RNAi-based mechanism for programmed genome rearrangements. Annu. Rev. Genet. 2005; 39:537–559. 16285871 10.1146/annurev.genet.39.073003.095906

[11] Yao M.-C., Chao J.-L., Cheng C.-Y. Programmed genome rearrangements in tetrahymena. Microbiol. Spectr. 2014; 2:doi:10.1128/microbiolspec.MDNA3-0012-2014. 10.1128/microbiolspec.MDNA3-0012-2014 26104448

[12] Hamilton E.P., Kapusta A., Huvos P.E., Bidwell S.L., Zafar N., Tang H., Hadjithomas M., Krishnakumar V., Badger J.H., Caler E.V. et al. Structure of the germline genome of Tetrahymena thermophila and relationship to the massively rearranged somatic genome. eLife. 2016; 5:e19090. 27892853 10.7554/eLife.19090 PMC5182062

[13] Yao M.C., Gorovsky M.A. Comparison of the sequences of macro- and micronuclear DNA of Tetrahymena pyriformis. Chromosoma. 1974; 48:1–18. 4218159 10.1007/BF00284863

[14] Chalker D.L., Yao M.C. Nongenic, bidirectional transcription precedes and may promote developmental DNA deletion in Tetrahymena thermophila. Genes Dev. 2001; 15:1287–1298. 11358871 10.1101/gad.884601 PMC313804

[15] Malone C.D., Hannon G.J. Small RNAs as guardians of the genome. Cell. 2009; 136:656–668. 19239887 10.1016/j.cell.2009.01.045 PMC2792755

[16] Mochizuki K., Fine N.A., Fujisawa T., Gorovsky M.A. Analysis of a piwi-related gene implicates small RNAs in genome rearrangement in tetrahymena. Cell. 2002; 110:689–699. 12297043 10.1016/s0092-8674(02)00909-1

[17] Chalker D.L., Yao M.C. Communication between parental and developing genomes during tetrahymena nuclear differentiation is likely mediated by homologous RNAs. Genetics. 2004; 169:149–160. 15466428 10.1534/genetics.104.032300 PMC1448867

[18] Liu Y., Taverna S.D., Muratore T.L., Shabanowitz J., Hunt D.F., Allis C.D. RNAi-dependent H3K27 methylation is required for heterochromatin formation and DNA elimination in Tetrahymena. Genes Dev. 2007; 21:1530–1545. 17575054 10.1101/gad.1544207 PMC1891430

[19] Madireddi M.T., Coyne R.S., Smothers J.F., Mickey K.M., Yao M.C., Allis C.D. Pdd1p, a novel chromodomain-containing protein, links heterochromatin assembly and DNA elimination in Tetrahymena. Cell. 1996; 87:75–84. 8858150 10.1016/s0092-8674(00)81324-0

[20] Taverna S.D., Coyne R.S., Allis C.D. Methylation of histone h3 at lysine 9 targets programmed DNA elimination in tetrahymena. Cell. 2002; 110:701–711. 12297044 10.1016/s0092-8674(02)00941-8

[21] Cheng C.-Y., Vogt A., Mochizuki K., Yao M.-C. A domesticated *piggyBac* transposase plays key roles in heterochromatin dynamics and DNA cleavage during programmed DNA deletion in *Tetrahymena thermophila*. Mol. Biol. Cell. 2010; 21:1753–1762. 20357003 10.1091/mbc.E09-12-1079 PMC2869380

[22] Austerberry C.F., Snyder R.O., Yao M.C. Sequence microheterogeneity is generated at junctions of programmed DNA deletions in Tetrahymena thermophila. Nucleic Acids Res. 1989; 17:7263–7272. 2798093 10.1093/nar/17.18.7263 PMC334806

[23] Lin I.-T., Chao J.-L., Yao M.-C. An essential role for the DNA breakage-repair protein Ku80 in programmed DNA rearrangements in *Tetrahymena thermophila*. Mol. Biol. Cell. 2012; 23:2213–2225. 22513090 10.1091/mbc.E11-11-0952 PMC3364183

[24] Cheng C.-Y., Young J.M., Lin C.-Y.G., Chao J.-L., Malik H.S., Yao M.-C. The piggyBac transposon-derived genes *TPB1* and *TPB6* mediate essential transposon-like excision during the developmental rearrangement of key genes in *Tetrahymena thermophila*. Genes Dev. 2016; 30:2724–2736. 28087716 10.1101/gad.290460.116 PMC5238731

[25] Feng L., Wang G., Hamilton E.P., Xiong J., Yan G., Chen K., Chen X., Dui W., Plemens A., Khadr L. et al. A germline-limited piggyBac transposase gene is required for precise excision in Tetrahymena genome rearrangement. Nucleic Acids Res. 2017; 45:9481–9502. 28934495 10.1093/nar/gkx652 PMC5766162

[26] Yao M.-C., Fuller P., Xi X. Programmed DNA deletion as an RNA-guided system of genome defense. Science. 2003; 300:1581–1584. 12791996 10.1126/science.1084737

[27] Austerberry C.F., Yao M.C. Sequence structures of two developmentally regulated, alternative DNA deletion junctions in Tetrahymena thermophila. Mol. Cell. Biol. 1988; 8:3947–3950. 3221871 10.1128/mcb.8.9.3947-3950.1988 PMC365456

[28] Yao M.-C., Choi J., Yokoyama S., Austerberry C.F., Yao C.-H. DNA elimination in tetrahymena: a developmental process involving extensive breakage and rejoining of DNA at defined sites. Cell. 1984; 36:433–440. 6319023 10.1016/0092-8674(84)90236-8

[29] Godiska R., Yao M.C. A programmed site-specific DNA rearrangement in Tetrahymena thermophila requires flanking polypurine tracts. Cell. 1990; 61:1237–1246. 2364428 10.1016/0092-8674(90)90688-b

[30] Godiska R., James C., Yao M.C. A distant 10-bp sequence specifies the boundaries of a programmed DNA deletion in Tetrahymena. Genes Dev. 1993; 7:2357–2365. 8253382 10.1101/gad.7.12a.2357

[31] Carle C.M., Zaher H.S., Chalker D.L. A parallel G quadruplex-binding protein regulates the boundaries of DNA elimination events of Tetrahymena thermophila. PLos Genet. 2016; 12:e1005842. 26950070 10.1371/journal.pgen.1005842 PMC4780704

[32] Chalker D.L., La Terza A., Wilson A., Kroenke C.D., Yao M.-C. Flanking regulatory sequences of the *Tetrahymena* R deletion element determine the boundaries of DNA rearrangement. Mol. Cell. Biol. 1999; 19:5631–5641. 10409752 10.1128/mcb.19.8.5631 PMC84415

[33] Fillingham J.S., Bruno D., Pearlman R.E. Cis-acting requirements in flanking DNA for the programmed elimination of mse2.9: a common mechanism for deletion of internal eliminated sequences from the developing macronucleus of Tetrahymena thermophila. Nucleic Acids Res. 2001; 29:488–498. 11139619 10.1093/nar/29.2.488 PMC29677

[34] Patil N.S., Karrer K.M. A developmentally regulated deletion element with long terminal repeats has cis-acting sequences in the flanking DNA. Nucleic Acids Res. 2000; 28:1465–1472. 10684943 10.1093/nar/28.6.1465 PMC111045

[35] Patil N.S., Hempen P.M., Udani R.A., Karrer K.M. Alternate junctions and microheterogeneity of Tlr1, a developmentally regulated DNA rearrangement in Tetrahymena thermophila. J. Eukaryot. Microbiol. 1997; 44:518–522. 9304822 10.1111/j.1550-7408.1997.tb05733.x

[36] Wells J.M., Ellingson J.L., Catt D.M., Berger P.J., Karrer K.M. A small family of elements with long inverted repeats is located near sites of developmentally regulated DNA rearrangement in Tetrahymena thermophila. Mol. Cell. Biol. 1994; 14:5939–5949. 8065327 10.1128/mcb.14.9.5939-5949.1994 PMC359120

[37] Eisen J.A., Coyne R.S., Wu M., Wu D., Thiagarajan M., Wortman J.R., Badger J.H., Ren Q., Amedeo P., Jones K.M. et al. Macronuclear genome sequence of the ciliate Tetrahymena thermophila, a model eukaryote. PLoS Biol. 2006; 4:e286. 16933976 10.1371/journal.pbio.0040286 PMC1557398

[38] Gorovsky M.A., Yao M.C., Keevert J.B., Pleger G.L. Isolation of micro- and macronuclei of Tetrahymena pyriformis. Methods Cell Biol. 1975; 9:311–327. 805898 10.1016/s0091-679x(08)60080-1

[39] Austerberry C.F., Yao M.C. Nucleotide sequence structure and consistency of a developmentally regulated DNA deletion in Tetrahymena thermophila. Mol. Cell. Biol. 1987; 7:435–443. 3031472 10.1128/mcb.7.1.435-443.1987 PMC365086

[40] Liu Y., Schröder J., Schmidt B. Musket: a multistage k-mer spectrum-based error corrector for Illumina sequence data. Bioinformatics. 2013; 29:308–315. 23202746 10.1093/bioinformatics/bts690

[41] Li H., Durbin R. Fast and accurate short read alignment with Burrows-Wheeler transform. Bioinformatics. 2009; 25:1754–1760. 19451168 10.1093/bioinformatics/btp324 PMC2705234

[42] Li H., Handsaker B., Wysoker A., Fennell T., Ruan J., Homer N., Marth G., Abecasis G., Durbin R. 1000 Genome Project Data Processing Subgroup The Sequence Alignment/Map format and SAMtools. Bioinformatics. 2009; 25:2078–2079. 19505943 10.1093/bioinformatics/btp352 PMC2723002

[43] Thorvaldsdottir H., Robinson J.T., Mesirov J.P. Integrative Genomics Viewer (IGV): high-performance genomics data visualization and exploration. Brief. Bioinform. 2013; 14:178–192. 22517427 10.1093/bib/bbs017 PMC3603213

[44] Fan X., Abbott T.E., Larson D., Chen K. Bateman A, Pearson WR, Stein LD, Stormo GD, Yates JR BreakDancer: Identification of genomic structural variation from paired-end read mapping: BreakDancer: Identification of genomic structural variation. Current Protocols in Bioinformatics. 2014; Hoboken John Wiley & Sons, Inc 15.6.1–15.6.11. 10.1002/0471250953.bi1506s45 PMC4138716 25152801

[45] Lin C.-Y.G., Lin I.-T., Yao M.-C. Programmed minichromosome elimination as a mechanism for somatic genome reduction in Tetrahymena thermophila. PLos Genet. 2016; 12:e1006403. 27806059 10.1371/journal.pgen.1006403 PMC5091840

[46] Chen C.-Y., Tsai H.-K., Hsu C.-M., May Chen M.-J., Hung H.-G., Huang G.T.-W., Li W.-H. Discovering gapped binding sites of yeast transcription factors. Proc. Natl. Acad. Sci. U.S.A. 2008; 105:2527–2532. 18272477 10.1073/pnas.0712188105 PMC2268170

[47] Bailey T.L., Williams N., Misleh C., Li W.W. MEME: discovering and analyzing DNA and protein sequence motifs. Nucleic Acids Res. 2006; 34:W369–W373. 16845028 10.1093/nar/gkl198 PMC1538909

[48] Austerberry C.F., Allis C.D., Yao M.C. Specific DNA rearrangements in synchronously developing nuclei of Tetrahymena. Proc. Natl. Acad. Sci. U.S.A. 1984; 81:7383–7387. 6095290 10.1073/pnas.81.23.7383 PMC392150

[49] Gros J., Rosu F., Amrane S., De Cian A., Gabelica V., Lacroix L., Mergny J.L. Guanines are a quartet's best friend: impact of base substitutions on the kinetics and stability of Tetramolecular quadruplexes. Nucleic Acids Res. 2007; 35:3064–3075. 17452368 10.1093/nar/gkm111 PMC1888817

[50] Schatz D.G., Ji Y. Recombination centres and the orchestration of V(D)J recombination. Nat. Rev. Immunol. 2011; 11:251–263. 21394103 10.1038/nri2941

