# Setting boundaries for genome-wide heterochromatic DNA deletions through flanking inverted repeats in *Tetrahymena thermophile*

**DOI:** 10.1093/nar/gkz481

**Published:** 2019-06-05

**Authors:** Chih-Yi Gabriela Lin, Ju-Lan Chao, Huai-Kuang Tsai, Douglas Chalker, Meng-Chao Yao

**Affiliations:** 1 Institute of Molecular Biology, Academia Sinica, 11529 Taipei, Taiwan; 2 Genome and Systems Biology Degree Program, National Taiwan University, 10617 Taipei, Taiwan; 3 Institute of Information Science, Academia Sinica, 11529 Taipei, Taiwan; 4 Department of Biology, Washington University in St. Louis, St. Louis, MO 63130, USA

*Nucleic Acids Research*, gkz209, https://doi.org/10.1093/nar/gkz209

The Authors wish to correct two errors in Figure 3 part I:

- The deletion line for Tm28, is misplaced. It was shifted to the right.
- The scale bar for this figure should be 100 bp, not 500 bp as shown.

**Figure 3.**
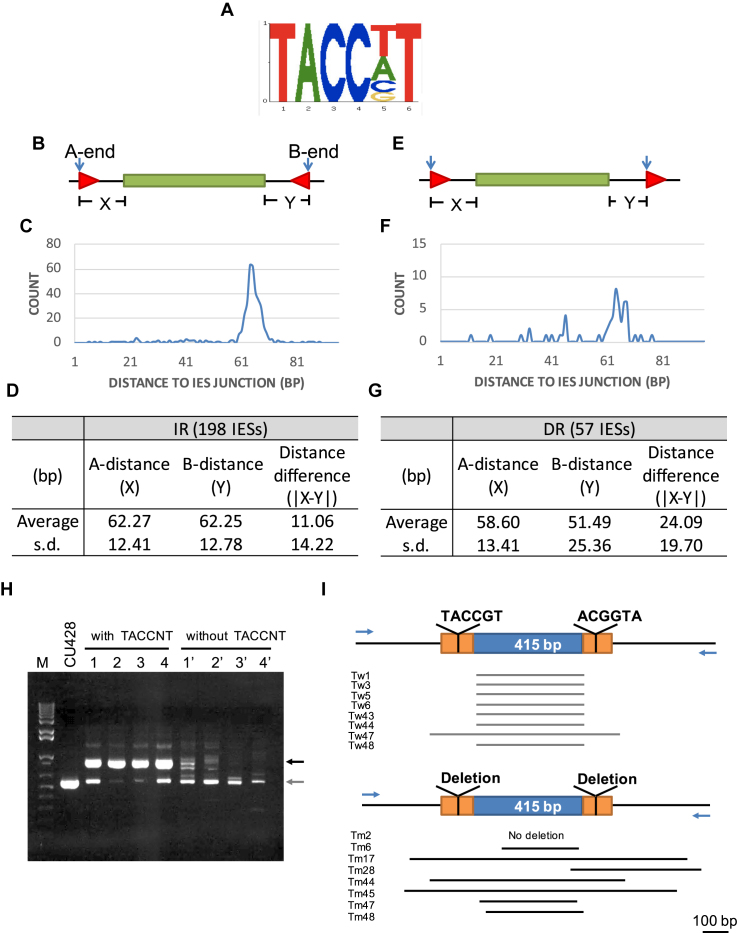
IR of the motif ‘TACCNT’ at similar distance to both ends of IESs. (A) Conserved sequence of ‘TACCNT’. (B) A cartoon shows the arrangement of IR that flanks an IES. (C) Tight distance distribution of the motifs as IRs near IESs in the CU427 genome. (D) Statistic information of the ‘TACCNT’ IRs in CU427. (E) A cartoon shows the arrangement of DR. (F) Distance distribution of the motifs as DRs near IESs in CU427. (G) Statistic information of the ‘TACCNT’ DRs in CU427. A-distance: distance of motif to one end of the IES; B-distance: distance of motif to the other end of the IES; distance difference: difference of the distances of the motif to either end of the IES; s.d.: standard deviation. (H) PCR of genomic DNA isolated from clones of IESs with or without the flanking T-domain. Dark arrow: expect arranged form; gray arrow: unspecific band. (I) Diagram of IES regions based on the sequencing result. Blue arrow: position of the primer set. Tw: single clone of WT IES with T-domain; Tm: single clone of mutated IES without T-domain. Arrow: primer.

These errors occurred during the preparation of the figure and do not affect the results or conclusions of the article. A new Figure 3 is provided below and the published article has been updated.

## Notes

Present address: Chih-Yi Gabriela Lin, Swiss Institute for Experimental Cancer Research, School of Life Sciences, Ecole Polytechnique Fédérale de Lausanne (EPFL), 1015 Lausanne, Switzerland.

