# Supplementary material for: Setting boundaries for genome-wide heterochromatic DNA deletions through flanking inverted repeats in Tetrahymena thermophila
Source: Nucleic Acids Res. 2019 Mar 28;47(10):5181–92. doi: 10.1093/nar/gkz209 (PMC6547420; doi:10.1093/nar/gkz209)
Supplement: gkz209_Supplemental_Files [file gkz209_supplemental_files.zip › Additional file 1_final.pdf]

Supplement Figure

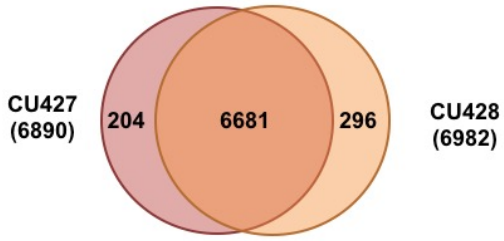

Interval	Number
0	1676
1-10	1792
11-20	645
21-30	246
31-40	196
41-50	168
51-60	139
61-70	116
71-80	77
81-90	84
91-100	81
>100	1461

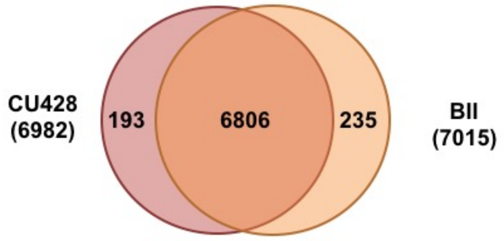

Interval	Number
0	1713
1-10	1880
11-20	662
21-30	273
31-40	186
41-50	157
51-60	114
61-70	120
71-80	81
81-90	72
91-100	66
>100	1387

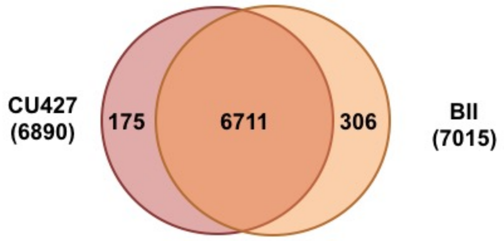

Interval	Number
0	1691
1-10	1920
11-20	758
21-30	308
31-40	225
41-50	188
51-60	132
61-70	131
71-80	101
81-90	82
91-100	77
>100	1193

Figure S1. IES shared between any two of the three inbred strains.

The Venn diagrams indicate the overlap between CU427, CU428 and BII. The table indicates the number of IESs in different intervals of the maximum boundary variations that overlapped between two strains.

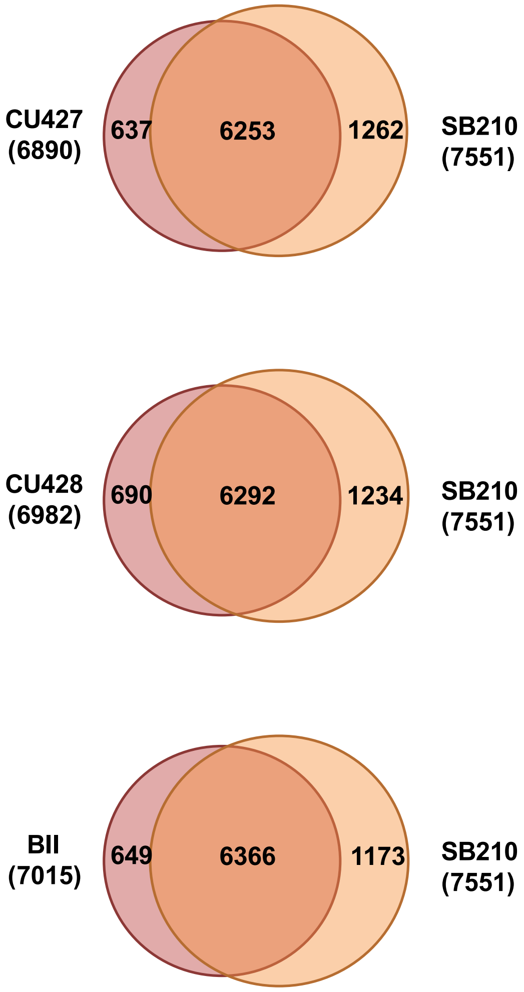

Figure S2. IES shared between the three inbreed strains and SB210

The Venn diagrams indicate the overlap between SB210 and CU427, CU428 or BII, respectively.

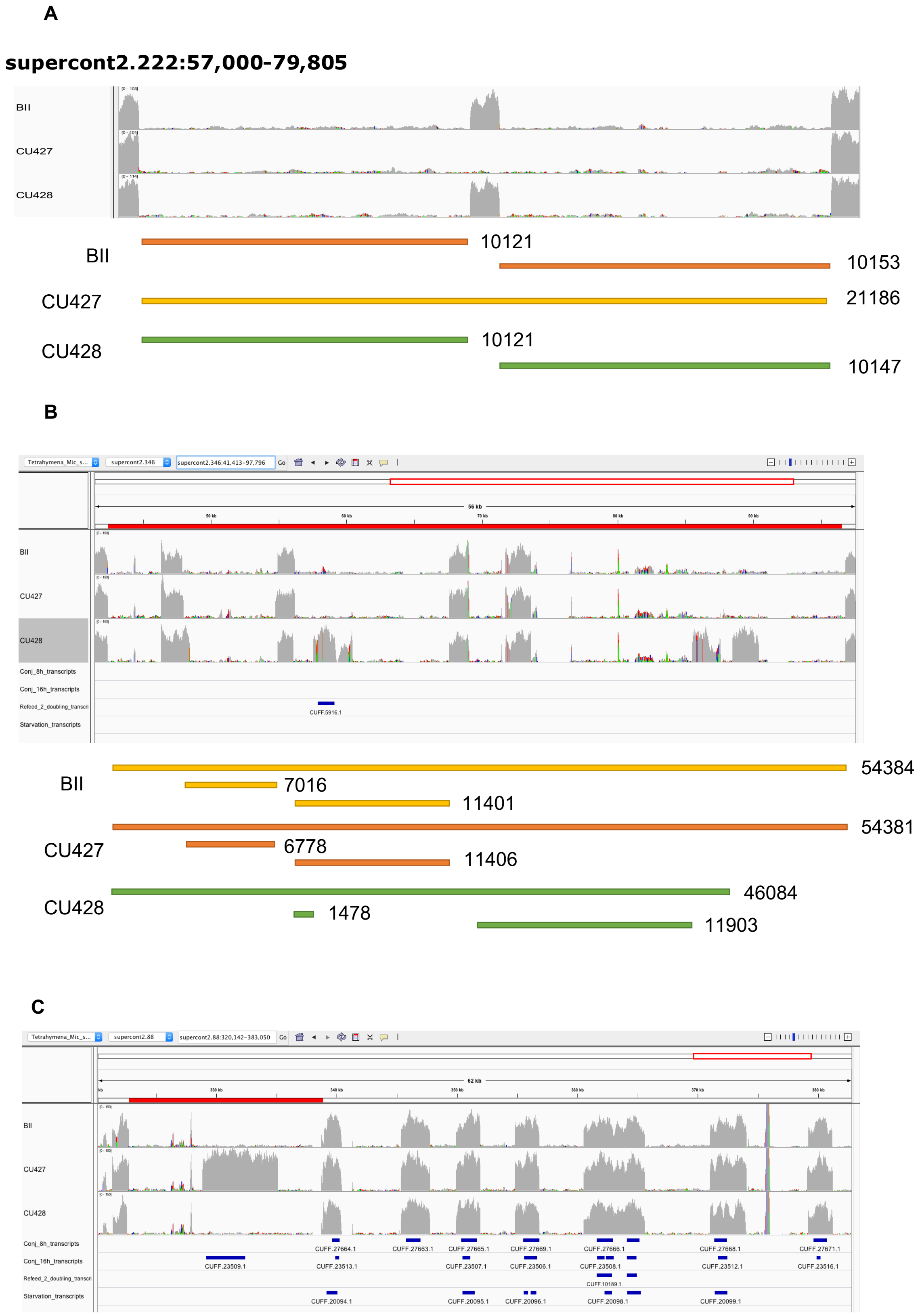

Figure S3. IES shows multiple forms at one location. (A) Location that contains 5 IES forms. (B) Location that contains 9 IES forms. (C) IES location and its nearby

region. Gray region: reads from the MAC genome; Red bar: IES location; blue bar: transcript; Conj_8h_transcripts: assembled transcripts at 8 hours post conjugation; Conj_16h_transcripts: assembled transcripts at 16 hours post conjugation; Refeed_2_doubling_transcripts: assembled transcripts at 2 doubling after refeeding; Starvation_transcripts: assembled transcripts during starvation.

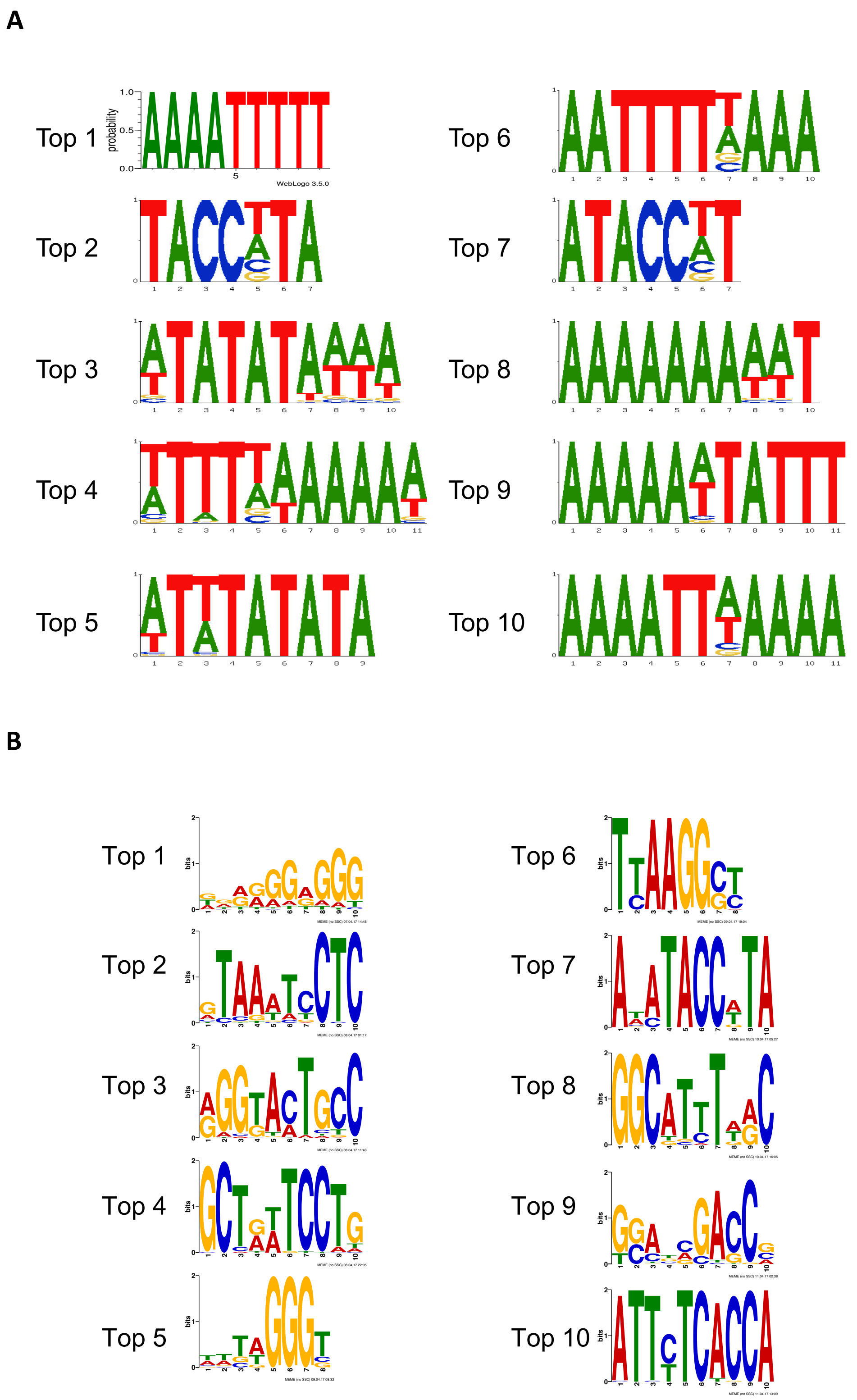

Figure S4. Motif mining of the entire collection of IES flanking regions.

(A) The top 10 motifs predicted using eTFBS. (B) The top 10 motifs predicted

using MEME.

A

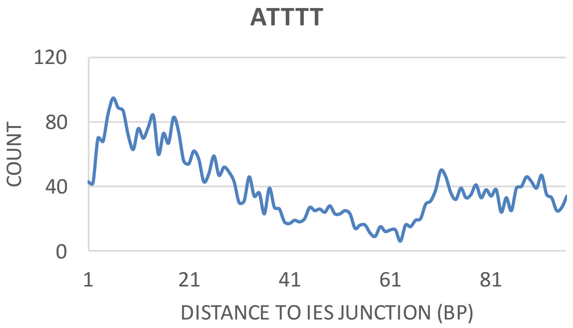

B

IR	Count	Average (bp)	Start (bp)	Q1 (bp)	Medium (bp) (Count)	Q3 (bp)	End (bp)	IQR (bp)	Maximum (bp) (Count)
ATTTT	3794	39.66	1	14	29 (49)	70	96	56	6 (95)

Figure S5. Distributions of "ATTTT" IRs within IES flanking regions in CU427.

(A) Distance distribution of "ATTTT" IRs. (B) Statistical data of "ATTTT" IRs.

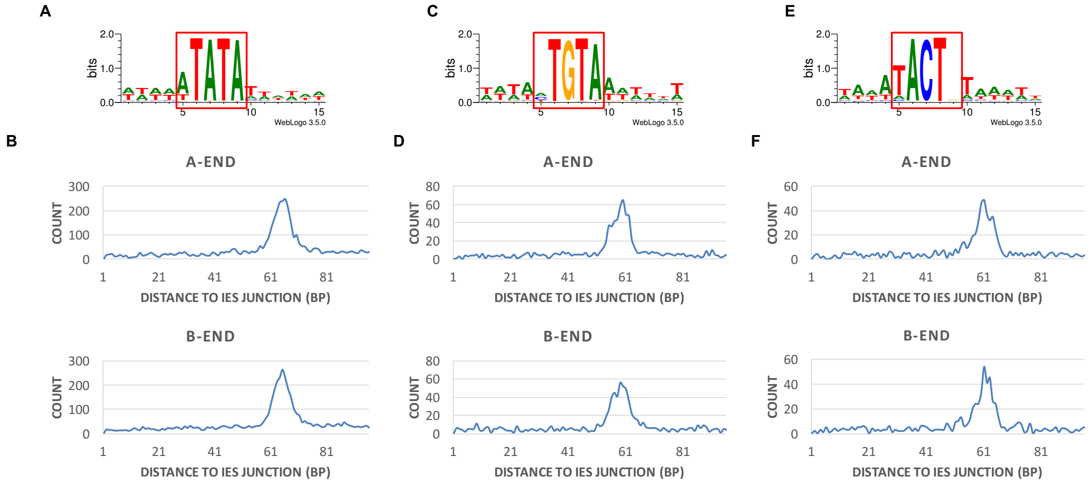

Figure S6. Distributions of pentamer IRs within IES flanking regions in CU427.

(A) and (B) Distribution of 2700 pentamer IRs that shared the same "TATA" sequence. (C) and (D) Distribution of 627 pentamer IRs that shared the same "TGTA" sequence. (E) and (F) Distribution of 504 pentamer IRs that shared the same "ACT" sequence. Note that the tight distance distributions of each copy to the IES boundary is not due to artificial restriction in (B), (D) and (F).

A

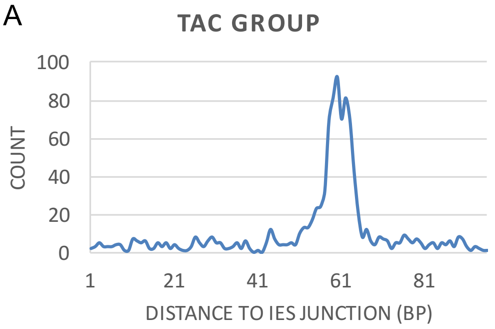

B

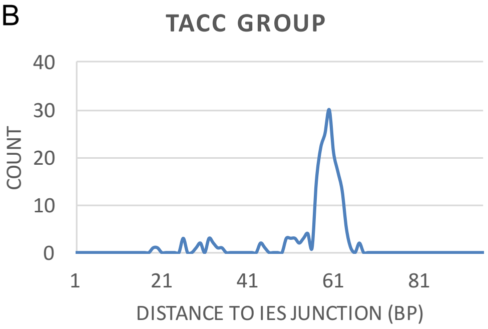

C

Sequence	Count	Average (bp)	Start (bp)	Q1 (bp)	Medium (bp) (Count)	Q3 (bp)	End (bp)	IQR (bp)	Maximum (bp) (Count)
TAC	1003	56.23	1	55	60 (92)	63	96	8	60 (92)
TACC	188	56.66	19	57	59 (25)	61	67	4	60 (30)

D

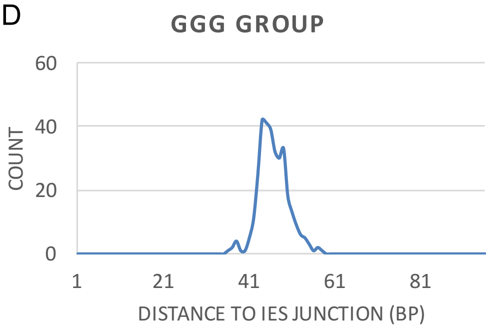

E

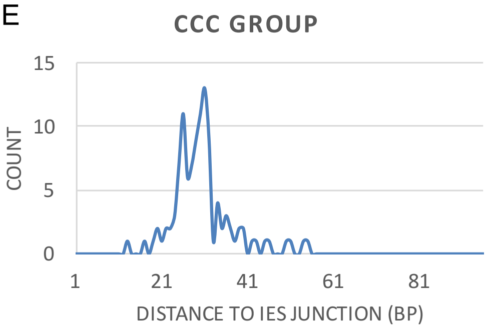

F

Sequence	Count	Average (bp)	Start (bp)	Q1 (bp)	Medium (bp) (Count)	Q3 (bp)	End (bp)	IQR (bp)	Maximum (bp) (Count)
GGG	325	46.59	36	44	46 (39)	49	58	5	44 (42)
CCC	111	30.41	13	26	30 (11)	32	55	6	31 (13)

Figure S7. Distributions of IRs within IES flanking regions in CU427. (A-C) Distributions of pentamer IRs with "TAC" and "TACC" within IES flanking regions in CU427. (D-F) Distributions of pentamer IRs with "GGG" and "CCC" within IES flanking regions in CU427.

A

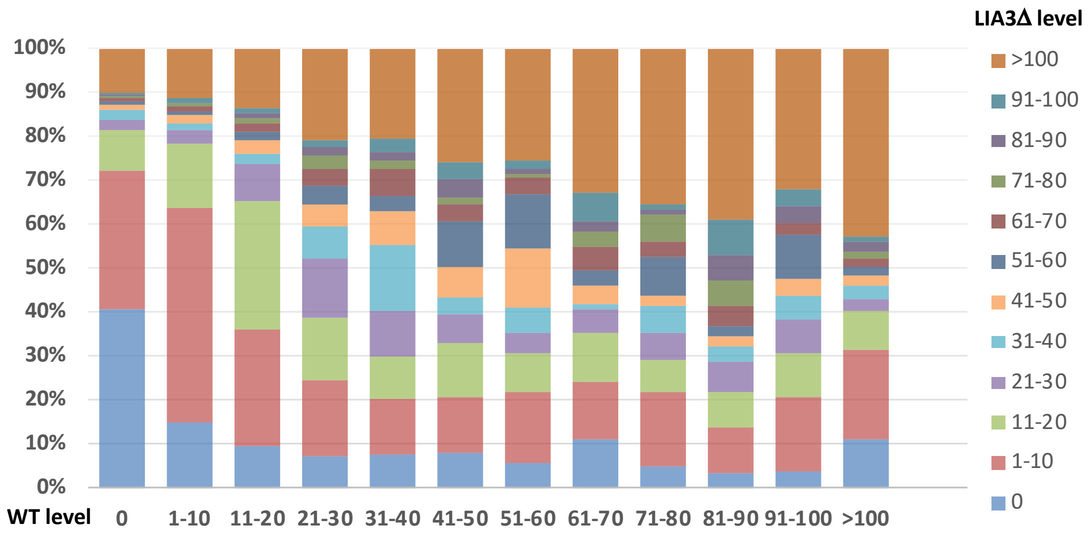

B

	WT												
		**0**	**1-10**	**11-20**	**21-30**	**31-40**	**41-50**	**51-60**	**61-70**	**71-80**	**81-90**	**91-100**	**>100**
	**0**	305	176	46	14	12	10	6	10	4	3	3	227
	**1-10**	240	578	126	33	20	16	17	12	14	9	13	408
	**11-20**	68	173	141	27	15	16	9	10	6	7	8	179
	**21-30**	18	36	41	26	16	8	5	5	5	6	6	59
	**31-40**	16	17	10	14	24	5	6	1	5	3	4	64
**LIA3Δ**	**41-50**	10	20	15	10	12	9	14	4	2	2	3	45
	**51-60**	5	13	10	8	5	13	13	3	7	2	8	41
	**61-70**	7	11	9	7	10	5	4	5	3	4	2	36
	**71-80**	2	10	5	6	3	2	1	3	5	5	0	33
	**81-90**	4	0	6	4	3	5	1	2	1	5	3	41
	**91-100**	2	14	5	3	5	5	2	6	1	7	3	29
	**>100**	77	133	66	40	32	33	27	30	29	34	25	867

Figure S8. (A) The histogram shows the number distribution of the IES variations among LIA3Δ strains according to the variations of these IESs in the WT dataset. (B) The comparison of the number of IESs in different bp variations among the WT

strains and among the LIA3 Δ strains.

Location	IES number	Boundary variation
Supercontig2.199: 150891-159601	3	63

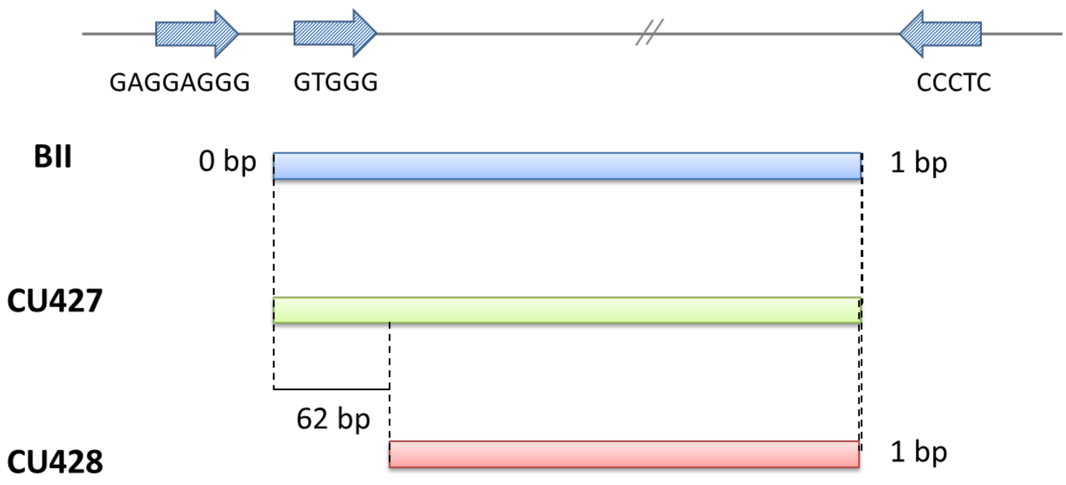

Figure S9. One case of alternative deletions of an IES.

This IES contains three deletion forms resulted from the alternative use of deletion boundaries. The left boundaries are identical between CU427 and BII and show a 62-bp difference between CU427 and CU428. The right boundaries have 1-bp variation in either combination. There are three copies of G-rich motifs near the IES boundaries, with two left copies to pair with one right copy to form IRs and all at similar distances to the respective boundaries. It suggests that the two pairs of IRs could both serve as the flanking regulatory elements for this IES.

Table S1. Validation of the predicted IESs against the published IESs.

		Published IES		Predicted IES			
	IES id	position1	position2	position1	position2	Difference among WT strains (bp)	Difference between check list and prediction (bp)
TPB1-dependent IES	regionF	1,281,326	1,281,461	1,281,326	1,281,461	0	0
	regionE	397,604	398,056	397,604	398,056	0	0
	regionC	178,349	178,536	178,349	178,536	0	0
	regionK	2,412,253	2,412,446	2,412,253	2,412,446	0	0
TPB2-dependent IES	M	120,714	121,617	120,711	121,615	321	5
	R	124,319	125,408	124,319	125,408	0	0
	Cam	90,604	91,983	90,605	91,988	0	6

* All of the terminal direct repeats (TDRs) are at the A-end of the IES. The A-end (position1) or B-end (position2) of IESs refer to the 5' and 3' end of the IES as it appears in the published MIC genome.

Table S2. IESs that contain the "TACCNT" IR at both ends of the IES flanking regions.

IES id	A-end position[1] (bp)	A-end sequence (bp)	B-end position[1] (bp)	B-end sequence[2] (bp)	Distance difference (bp)	Class interval[3] (bp)
IR						
CU427.Supercontig2.35.3423	62	TACCAT	65	TACCGT	3	0
CU427.Supercontig2.2.265	65	TACCAT	66	TACCAT	1	0
CU427.Supercontig2.4.584	67	TACCTT	62	TACCTT	5	0
CU427.Supercontig2.18.2055	66	TACCGT	64	TACCTT	2	0
CU427.Supercontig2.79.5595	65	TACCTT	67	TACCTT	2	0
CU427.Supercontig2.39.3643	60	TACCAT	68	TACCAT	8	0
CU427.Supercontig2.641.12089	65	TACCGT	66	TACCAT	1	0
CU427.Supercontig2.3.508	61	TACCAT	68	TACCGT	7	0
CU427.Supercontig2.340.10548	89	TACCCT	66	TACCTT	23	0
CU427.Supercontig2.4.615	66	TACCTT	66	TACCTT	0	0
CU427.Supercontig2.1.119	10	TACCTT	63	TACCTT	53	0
CU427.Supercontig2.406.11096	67	TACCAT	64	TACCAT	3	0
CU427.Supercontig2.26.2775	64	TACCTT	64	TACCTT	0	0
CU427.Supercontig2.32.3213	65	TACCGT	66	TACCGT	1	0
CU427.Supercontig2.3.512	67	TACCAT	60	TACCGT	7	0
CU427.Supercontig2.115.6897	6	TACCCT	66	TACCTT	60	0
CU427.Supercontig2.66.5092	19	TACCCT	60	TACCTT	41	0
CU427.Supercontig2.33.3321	64	TACCGT	64	TACCAT	0	0
CU427.Supercontig2.69.5208	70	TACCAT	63	TACCGT	7	0
CU427.Supercontig2.31.3179	67	TACCTT	23	TACCTT	44	0
CU427.Supercontig2.89.6054	64	TACCGT	66	TACCGT	2	0
CU427.Supercontig2.44.4010	69	TACCTT	61	TACCTT	8	0
CU427.Supercontig2.86.5930	68	TACCTT	15	TACCCT	53	0
CU427.Supercontig2.110.6718	66	TACCGT	63	TACCGT	3	0
CU427.Supercontig2.186.8605	75	TACCTT	65	TACCGT	10	0
CU427.Supercontig2.2.363	65	TACCAT	66	TACCGT	1	0
CU427.Supercontig2.9.1157	68	TACCGT	62	TACCTT	6	0
CU427.Supercontig2.36.3482	64	TACCCT	42	TACCAT	22	0
CU427.Supercontig2.6.911	72	TACCCT	65	TACCAT	7	0
CU427.Supercontig2.8.1031	53	TACCTT	70	TACCAT	17	0
CU427.Supercontig2.131.7363	40	TACCTT	65	TACCAT	25	0
CU427.Supercontig2.170.8281	70	TACCGT	63	TACCGT	7	0
CU427.Supercontig2.134.7444	27	TACCAT	64	TACCCT	37	0
CU427.Supercontig2.7.979	63	TACCAT	66	TACCTT	3	0
CU427.Supercontig2.416.11162	63	TACCTT	61	TACCAT	2	0
CU427.Supercontig2.283.10000	64	TACCTT	35	TACCCT	29	0
CU427.Supercontig2.13.1698	64	TACCTT	64	TACCTT	0	0
CU427.Supercontig2.86.5910	64	TACCAT	67	TACCTT	3	0
CU427.Supercontig2.128.7297	63	TACCCT	67	TACCGT	4	0
CU427.Supercontig2.152.7912	57	TACCCT	65	TACCTT	8	0
CU427.Supercontig2.694.12158	68	TACCTT	64	TACCAT	4	0
CU427.Supercontig2.213.9047	68	TACCGT	63	TACCGT	5	0
CU427.Supercontig2.2.299	62	TACCTT	70	TACCTT	8	0
CU427.Supercontig2.626.12060	63	TACCTT	66	TACCGT	3	0

CU427.Supercontig2.424.11222	64	TACCAT	67	TACCTT	3	0
CU427.Supercontig2.42.3933	57	TACCTT	67	TACCTT	10	0
CU427.Supercontig2.423.11210	68	TACCTT	63	TACCTT	5	0
CU427.Supercontig2.235.9388	35	TACCCT	38	TACCCT	3	0
CU427.Supercontig2.62.4906	62	TACCAT	68	TACCGT	6	0
CU427.Supercontig2.199.8832	68	TACCAT	64	TACCGT	4	0
CU427.Supercontig2.70.5216	44	TACCAT	74	TACCAT	30	0
CU427.Supercontig2.180.8490	68	TACCTT	65	TACCAT	3	0
CU427.Supercontig2.34.3353	43	TACCAT	65	TACCGT	22	0
CU427.Supercontig2.3.426	39	TACCCT	14	TACCAT	25	0
CU427.Supercontig2.104.6522	64	TACCTT	64	TACCAT	0	1
CU427.Supercontig2.107.6643	65	TACCAT	64	TACCGT	1	1
CU427.Supercontig2.217.9127	66	TACCAT	23	TACCAT	43	1
CU427.Supercontig2.72.5322	46	TACCTT	65	TACCTT	19	1
CU427.Supercontig2.24.2558	65	TACCAT	67	TACCAT	2	1
CU427.Supercontig2.112.6766	67	TACCCT	67	TACCAT	0	1
CU427.Supercontig2.335.10494	64	TACCAT	65	TACCAT	1	1
CU427.Supercontig2.310.10241	64	TACCGT	65	TACCGT	1	1
CU427.Supercontig2.332.10473	43	TACCAT	68	TACCTT	25	1
CU427.Supercontig2.51.4417	60	TACCAT	66	TACCTT	6	1
CU427.Supercontig2.194.8744	67	TACCAT	62	TACCCT	5	1
CU427.Supercontig2.56.4648	67	TACCAT	64	TACCTT	3	1
CU427.Supercontig2.561.11903	65	TACCGT	67	TACCGT	2	1
CU427.Supercontig2.21.2408	63	TACCGT	68	TACCGT	5	1
CU427.Supercontig2.1.143	68	TACCTT	64	TACCAT	4	1
CU427.Supercontig2.9.1223	63	TACCTT	65	TACCTT	2	1
CU427.Supercontig2.9.1152	64	TACCAT	63	TACCAT	1	1
CU427.Supercontig2.82.5717	69	TACCCT	64	TACCAT	5	1
CU427.Supercontig2.452.11407	76	TACCAT	64	TACCAT	12	1
CU427.Supercontig2.56.4620	63	TACCAT	65	TACCTT	2	1
CU427.Supercontig2.288.10053	69	TACCAT	63	TACCTT	6	1
CU427.Supercontig2.220.9203	64	TACCAT	69	TACCGT	5	1
CU427.Supercontig2.115.6903	62	TACCAT	66	TACCGT	4	1
CU427.Supercontig2.172.8323	52	TACCAT	65	TACCGT	13	1
CU427.Supercontig2.318.10337	63	TACCAT	65	TACCAT	2	1
CU427.Supercontig2.362.10729	68	TACCAT	83	TACCAT	15	1
CU427.Supercontig2.99.6368	66	TACCGT	64	TACCGT	2	1
CU427.Supercontig2.133.7421	64	TACCGT	68	TACCGT	4	1
CU427.Supercontig2.219.9184	67	TACCAT	65	TACCAT	2	1
CU427.Supercontig2.6.891	31	TACCAT	41	TACCTT	10	1
CU427.Supercontig2.29.3082	100	TACCTT	62	TACCGT	38	1
CU427.Supercontig2.62.4852	64	TACCTT	39	TACCTT	25	1
CU427.Supercontig2.65.5040	62	TACCGT	64	TACCTT	2	1
CU427.Supercontig2.211.9040	65	TACCTT	65	TACCAT	0	1
CU427.Supercontig2.405.11082	64	TACCAT	65	TACCAT	1	1
CU427.Supercontig2.415.11157	65	TACCAT	65	TACCAT	0	1
CU427.Supercontig2.126.7231	66	TACCAT	66	TACCAT	0	1
CU427.Supercontig2.96.6264	67	TACCAT	61	TACCGT	6	1
CU427.Supercontig2.150.7864	65	TACCGT	67	TACCAT	2	1
CU427.Supercontig2.205.8945	67	TACCCT	63	TACCAT	4	1
CU427.Supercontig2.391.10974	87	TACCAT	63	TACCCT	24	1
CU427.Supercontig2.50.4339	64	TACCAT	30	TACCAT	34	1
CU427.Supercontig2.219.9176	65	TACCTT	67	TACCGT	2	1
CU427.Supercontig2.302.10178	99	TACCAT	64	TACCAT	35	1
CU427.Supercontig2.147.7782	67	TACCGT	64	TACCTT	3	1

CU427.Supercontig2.18.2076	65	TACCTT	61	TACCAT	4	1
CU427.Supercontig2.34.3367	63	TACCAT	65	TACCAT	2	1
CU427.Supercontig2.53.4480	64	TACCTT	62	TACCTT	2	1
CU427.Supercontig2.97.6272	63	TACCAT	22	TACCAT	41	1
CU427.Supercontig2.136.7503	64	TACCGT	65	TACCGT	1	1
CU427.Supercontig2.132.7386	68	TACCTT	63	TACCAT	5	1
CU427.Supercontig2.140.7561	66	TACCGT	64	TACCGT	2	1
CU427.Supercontig2.118.7011	69	TACCGT	64	TACCGT	5	1
CU427.Supercontig2.408.11110	64	TACCAT	66	TACCGT	2	1
CU427.Supercontig2.36.3537	64	TACCTT	67	TACCAT	3	1
CU427.Supercontig2.384.10902	65	TACCGT	64	TACCTT	1	1
CU427.Supercontig2.4.603	64	TACCAT	62	TACCGT	2	1
CU427.Supercontig2.68.5163	98	TACCAT	66	TACCAT	32	1
CU427.Supercontig2.570.11932	66	TACCAT	64	TACCTT	2	1
CU427.Supercontig2.341.10549	65	TACCTT	67	TACCGT	2	1
CU427.Supercontig2.43.3978	41	TACCCT	63	TACCAT	22	1
CU427.Supercontig2.688.12151	62	TACCAT	65	TACCCT	3	1
CU427.Supercontig2.43.3953	66	TACCGT	66	TACCGT	0	1
CU427.Supercontig2.10.1409	69	TACCGT	61	TACCGT	8	1
CU427.Supercontig2.2.323	69	TACCAT	62	TACCTT	7	1
CU427.Supercontig2.71.5279	48	TACCTT	98	TACCAT	50	1
CU427.Supercontig2.1.92	65	TACCAT	64	TACCAT	1	1
CU427.Supercontig2.40.3766	41	TACCCT	61	TACCAT	20	1
CU427.Supercontig2.4.598	63	TACCGT	68	TACCAT	5	1
CU427.Supercontig2.77.5497	66	TACCGT	67	TACCAT	1	1
CU427.Supercontig2.164.8179	46	TACCGT	61	TACCGT	15	1
CU427.Supercontig2.414.11150	68	TACCAT	62	TACCTT	6	1
CU427.Supercontig2.2.330	49	TACCTT	67	TACCTT	18	1
CU427.Supercontig2.3.524	63	TACCAT	69	TACCTT	6	1
CU427.Supercontig2.25.2599	50	TACCAT	64	TACCAT	14	1
CU427.Supercontig2.507.11706	63	TACCAT	71	TACCAT	8	1
CU427.Supercontig2.108.6653	66	TACCTT	65	TACCTT	1	1
CU427.Supercontig2.132.7390	69	TACCTT	62	TACCAT	7	1
CU427.Supercontig2.229.9301	65	TACCGT	66	TACCGT	1	1
CU427.Supercontig2.469.11521	66	TACCAT	65	TACCTT	1	1
CU427.Supercontig2.50.4340	63	TACCAT	66	TACCAT	3	1
CU427.Supercontig2.50.4322	64	TACCGT	67	TACCAT	3	1
CU427.Supercontig2.330.10456	64	TACCTT	62	TACCAT	2	1
CU427.Supercontig2.174.8368	66	TACCAT	61	TACCAT	5	1
CU427.Supercontig2.23.2519	42	TACCTT	65	TACCTT	23	1
CU427.Supercontig2.11.1472	65	TACCTT	24	TACCCT	41	11
CU427.Supercontig2.668.12125	64	TACCAT	67	TACCCT	3	11
CU427.Supercontig2.94.6191	65	TACCAT	85	TACCTT	20	11
CU427.Supercontig2.52.4422	68	TACCAT	65	TACCAT	3	11
CU427.Supercontig2.40.3765	66	TACCGT	63	TACCGT	3	11
CU427.Supercontig2.273.9889	62	TACCCT	48	TACCAT	14	11
CU427.Supercontig2.124.7183	61	TACCTT	70	TACCCT	9	11
CU427.Supercontig2.222.9224	69	TACCAT	63	TACCCT	6	11
CU427.Supercontig2.7.1017	74	TACCAT	17	TACCCT	57	11
CU427.Supercontig2.104.6548	72	TACCGT	65	TACCAT	7	11
CU427.Supercontig2.279.9962	86	TACCTT	67	TACCTT	19	11
CU427.Supercontig2.54.4537	65	TACCTT	65	TACCAT	0	11
CU427.Supercontig2.73.5347	66	TACCGT	63	TACCGT	3	11
CU427.Supercontig2.110.6736	64	TACCTT	71	TACCAT	7	11
CU427.Supercontig2.339.10534	64	TACCTT	70	TACCAT	6	11

CU427.Supercontig2.421.11197	66	TACCGT	62	TACCGT	4	11
CU427.Supercontig2.261.9748	63	TACCAT	79	TACCAT	16	11
CU427.Supercontig2.347.10610	58	TACCGT	72	TACCGT	14	11
CU427.Supercontig2.63.4915	70	TACCCT	18	TACCCT	52	11
CU427.Supercontig2.4.580	69	TACCGT	62	TACCGT	7	11
CU427.Supercontig2.279.9960	21	TACCCT	66	TACCGT	45	11
CU427.Supercontig2.76.5479	64	TACCAT	66	TACCAT	2	21
CU427.Supercontig2.34.3359	44	TACCTT	62	TACCAT	18	21
CU427.Supercontig2.98.6294	68	TACCGT	67	TACCGT	1	21
CU427.Supercontig2.133.7426	64	TACCTT	65	TACCAT	1	21
CU427.Supercontig2.47.4176	65	TACCAT	60	TACCTT	5	21
CU427.Supercontig2.11.1498	63	TACCTT	68	TACCTT	5	31
CU427.Supercontig2.86.5926	69	TACCTT	24	TACCTT	45	41
CU427.Supercontig2.61.4848	64	TACCAT	65	TACCTT	1	41
CU427.Supercontig2.303.10184	65	TACCAT	98	TACCAT	33	51
CU427.Supercontig2.107.6632	66	TACCAT	64	TACCAT	2	91
CU427.Supercontig2.78.5542	60	TACCTT	65	TACCTT	5	151
CU427.Supercontig2.98.6312	29	TACCTT	66	TACCGT	37	181
CU427.Supercontig2.494.11621	33	TACCAT	64	TACCGT	31	191
CU427.Supercontig2.33.3320	65	TACCGT	67	TACCAT	2	191
CU427.Supercontig2.9.1169	67	TACCAT	37	TACCCT	30	211
CU427.Supercontig2.109.6699	67	TACCTT	68	TACCTT	1	211
CU427.Supercontig2.3.400	65	TACCAT	65	TACCAT	0	231
CU427.Supercontig2.27.2815	65	TACCAT	64	TACCAT	1	251
CU427.Supercontig2.206.8965	64	TACCAT	65	TACCCT	1	281
CU427.Supercontig2.514.11737	68	TACCGT	64	TACCTT	4	291
CU427.Supercontig2.26.2676	67	TACCAT	64	TACCAT	3	301
CU427.Supercontig2.13.1722	65	TACCAT	66	TACCAT	1	311
CU427.Supercontig2.99.6342	65	TACCCT	97	TACCAT	32	321
CU427.Supercontig2.544.11851	62	TACCTT	71	TACCTT	9	321
CU427.Supercontig2.274.9896	68	TACCGT	63	TACCGT	5	331
CU427.Supercontig2.41.3797	58	TACCTT	68	TACCAT	10	331
CU427.Supercontig2.7.964	68	TACCTT	8	TACCTT	60	351
CU427.Supercontig2.41.3787	70	TACCCT	63	TACCTT	7	391
CU427.Supercontig2.69.5176	62	TACCAT	68	TACCGT	6	411
CU427.Supercontig2.22.2428	64	TACCAT	66	TACCGT	2	411
CU427.Supercontig2.30.3089	23	TACCTT	64	TACCAT	41	431
CU427.Supercontig2.100.6381	64	TACCGT	69	TACCTT	5	491
CU427.Supercontig2.237.9422	67	TACCGT	60	TACCGT	7	551
CU427.Supercontig2.113.6808	23	TACCTT	68	TACCTT	45	611
CU427.Supercontig2.571.11935	67	TACCAT	62	TACCCT	5	721
CU427.Supercontig2.14.1794	65	TACCGT	65	TACCCT	0	771
CU427.Supercontig2.33.3282	66	TACCGT	31	TACCTT	35	6321
CU427.Supercontig2.153.7928	16	TACCTT	69	TACCTT	53	14041
CU427.Supercontig2.222.9221	65	TACCAT	68	TACCCT	3	22081
DR						
CU427.Supercontig2.641.12089	65	TACCGT	95	TACCTT	30	0
CU427.Supercontig2.66.5092	19	TACCCT	97	TACCTT	78	0
CU427.Supercontig2.70.5218	46	TACCTT	60	TACCCT	14	0
CU427.Supercontig2.86.5930	68	TACCTT	64	TACCTT	4	0
CU427.Supercontig2.535.11809	64	TACCTT	9	TACCTT	55	0
CU427.Supercontig2.28.2958	65	TACCTT	97	TACCAT	32	0
CU427.Supercontig2.194.8735	66	TACCGT	97	TACCCT	31	0
CU427.Supercontig2.203.8900	64	TACCGT	85	TACCTT	21	0
CU427.Supercontig2.220.9197	71	TACCTT	48	TACCCT	23	0

CU427.Supercontig2.180.8490	68	TACCTT	62	TACCTT	6	0
CU427.Supercontig2.131.7375	63	TACCAT	57	TACCCT	6	1
CU427.Supercontig2.473.11553	65	TACCAT	41	TACCTT	24	1
CU427.Supercontig2.1.143	68	TACCTT	10	TACCAT	58	1
CU427.Supercontig2.9.1223	63	TACCTT	37	TACCGT	26	1
CU427.Supercontig2.61.4841	62	TACCAT	11	TACCTT	51	1
CU427.Supercontig2.1.4	61	TACCGT	65	TACCCT	4	1
CU427.Supercontig2.69.5173	64	TACCCT	57	TACCTT	7	1
CU427.Supercontig2.54.4512	67	TACCAT	21	TACCAT	46	1
CU427.Supercontig2.415.11157	65	TACCAT	83	TACCTT	18	1
CU427.Supercontig2.40.3744	12	TACCTT	52	TACCCT	40	1
CU427.Supercontig2.50.4339	64	TACCAT	50	TACCTT	14	1
CU427.Supercontig2.430.11255	77	TACCAT	10	TACCAT	67	1
CU427.Supercontig2.311.10257	68	TACCTT	67	TACCTT	1	1
CU427.Supercontig2.89.6055	64	TACCAT	13	TACCTT	51	1
CU427.Supercontig2.4.603	41	TACCCT	40	TACCTT	1	1
CU427.Supercontig2.19.2197	66	TACCAT	77	TACCCT	11	1
CU427.Supercontig2.28.2955	64	TACCAT	66	TACCTT	2	1
CU427.Supercontig2.28.2972	64	TACCTT	49	TACCCT	15	1
CU427.Supercontig2.50.4322	64	TACCGT	13	TACCTT	51	1
CU427.Supercontig2.224.9244	63	TACCTT	60	TACCTT	3	11
CU427.Supercontig2.40.3765	66	TACCGT	17	TACCTT	49	11
CU427.Supercontig2.276.9926	67	TACCAT	10	TACCAT	57	11
CU427.Supercontig2.162.8126	39	TACCTT	57	TACCAT	18	11
CU427.Supercontig2.53.4465	67	TACCAT	67	TACCCT	0	11
CU427.Supercontig2.194.8731	65	TACCGT	43	TACCTT	22	11
CU427.Supercontig2.25.2638	63	TACCCT	61	TACCTT	2	11
CU427.Supercontig2.203.8892	44	TACCTT	35	TACCAT	9	11
CU427.Supercontig2.140.7586	67	TACCAT	21	TACCTT	46	21
CU427.Supercontig2.545.11854	73	TACCAT	42	TACCAT	31	21
CU427.Supercontig2.640.12085	68	TACCTT	66	TACCTT	2	51
CU427.Supercontig2.443.11349	51	TACCAT	79	TACCAT	28	61
CU427.Supercontig2.224.9254	58	TACCCT	45	TACCTT	13	61
CU427.Supercontig2.63.4958	46	TACCCT	32	TACCCT	14	91
CU427.Supercontig2.214.9090	30	TACCCT	80	TACCCT	50	111
CU427.Supercontig2.494.11621	33	TACCAT	32	TACCTT	1	191
CU427.Supercontig2.259.9740	46	TACCCT	37	TACCTT	9	191
CU427.Supercontig2.2.289	62	TACCAT	71	TACCTT	9	191
CU427.Supercontig2.340.10543	45	TACCAT	49	TACCTT	4	201
CU427.Supercontig2.397.11024	46	TACCCT	59	TACCAT	13	251
CU427.Supercontig2.26.2676	67	TACCAT	14	TACCTT	53	301
CU427.Supercontig2.28.2959	33	TACCAT	56	TACCCT	23	341
CU427.Supercontig2.7.964	68	TACCTT	52	TACCTT	16	351
CU427.Supercontig2.361.10723	62	TACCCT	43	TACCCT	19	441
CU427.Supercontig2.48.4213	60	TACCCT	84	TACCTT	24	591
CU427.Supercontig2.36.3521	61	TACCGT	41	TACCAT	20	611
CU427.Supercontig2.571.11935	67	TACCAT	50	TACCCT	17	721
CU427.Supercontig2.363.10734	65	TACCGT	99	TACCAT	34	11021

[1] The position indicates the distance of the sequence to the IES boundary.

[2] The sequence in the B-end is presented as the reverse complement.

[3] We defined each 10 bp as an interval, except for 0 bp that is a separate group. The number indicates the minimum number of the representative interval.

Table S3. Position of the IES boundary of the TACCNT-absent IES clones

Clone	Position of FRS1	nt of BS1	Position of BS1	Positin of FRS2	nt of BS2	Position of BS2	Sequence of FRS	Distance to BS1	Distance to BS2
Tm6	Unknown	G	1538	Unknown	A	1825	Unknown	Unknown	Unknown
Tm17	1119	C	1170	2302	A	2253	CTGGG	51	49
Tm28	2202	T	2264	2361	C	2312	ATGCA	62	49
Tm44	1231	T	1263	2014	A	1996	AAGCAG	32	18
Tm45	1120	T	1166	2262	A	2209	TGGGT	46	53
Tm47	1401	T	1452	1881	A	1822	GGACT	51	59
Tm48	Unknown	G	1468	Unknown	T	1864	Unknown	Unknown	Unknown

* FRS: Flanking regulatory sequence; nt: nucleotide; BS: Breakage site

Table S4. List of trimer, tetramer and pentamer IRs within IES flanking regions (IQR ≤ 10).

Group	Count	Average	Q0	Q1	medium	Q3	Q4	IQR	
TAC	1003	56.23	1	55	60(92)	63	96	8	
TGT	702	54.50	1	51	57(49)	61	96	10	
AGG	374	45.63	5	43	46(28)	49	89	6	
CTC	330	45.42	1	42	48(32)	51	90	9	
GGG	325	46.59	36	44	46(39)	49	58	5	
GGT	215	45.59	1	43	45(18)	48	96	5	
GGA	184	45.53	19	43	46(17)	49	67	6	
CTG	161	58.31	41	56	58(25)	60	95	4	
GAG	128	46.38	23	45	47(17)	49	82	4	
CCC	111	30.41	13	26	30(11)	32	55	6	
CCG	61	59.18	56	58	59(11)	60	64	2	
CAC	58	37.98	4	32	39(4)	41	67	9	
GTG	52	47.88	36	45	48(4)	50	61	5	
CGT	48	58.31	55	57	58(9)	59	63	2	
GGC	29	37.41	31	36	37(4)	39	44	3	
GTC	17	44.18	27	41	45(1)	50	56	9	
GAC	15	37.13	27	32	37(1)	39	48	7	
CGA	4	15.50	10	15	18(1)	19	19	4	
TATA	4119	61.63	1	59	63(292)	68	96	9	
ATAC	458	58.82	2	58	61(46)	63	91	5	
GTAA	363	54.21	1	52	56(31)	60	96	8	Two peaks, often with TGT
TACT	323	57.67	1	55	59(33)	62	96	7	
TGTA	307	56.32	1	54	57(32)	60	93	6	
TACC	188	56.66	19	57	59(25)	61	67	4	
AGGG	153	46.82	37	45	46(21)	49	57	4	
CTCA	145	46.72	11	45	48(17)	50	90	5	
ACTG	108	58.35	51	56	58(18)	60	69	4	
CTGT	104	58.08	41	56	58(19)	59	95	3	
GGGG	101	47.98	37	46	48(9)	50	58	4	
TCTC	99	48.34	29	43	49(8)	51	90	8	
GGGT	88	45.26	38	44	45(13)	47	51	3	
GAGG	78	46.91	38	45	47(13)	49	54	4	
TCCT	74	50.91	18	48	50(8)	53	88	5	
GGTA	73	45.26	21	42	44(9)	47	64	5	
ACCT	72	51.83	19	50	57(8)	60	64	10	
ACCA	70	55.40	23	56	58(16)	59	86	3	
CCAT	69	51.14	11	54	57(12)	58	61	4	
GGGA	62	45.31	36	43	45(7)	47	55	4	
CCCT	54	30.09	19	26	29(6)	31	55	5	

ATCC	53	50.28	13	49	51(6)	54	75	5
TAGG	53	44.96	9	45	48(4)	51	59	6
CTCT	48	46.35	23	45	49(2)	52	62	7
ACCC	47	33.04	23	29	31(4)	36	55	7
ACCG	46	59.57	56	58	59(10)	61	64	3
AGGA	45	46.42	35	41	47(6)	49	59	8
GGAG	43	46.91	33	46	47(8)	49	52	3
ACTC	42	47.21	1	46	50(4)	54	63	8
GGAT	38	44.97	26	41	46(5)	47	57	6
GGTT	38	40.76	1	41	44(2)	47	56	6
CCGT	37	58.68	56	57	58(8)	59	63	2
GTAG	33	49.09	41	45	47(2)	51	60	6
GGAA	32	46.19	22	42	45(3)	51	67	9
TGGG	31	47.00	43	45	46(5)	48	55	3
TTCC	31	47.39	5	48	50(3)	53	88	5
CCCC	28	29.89	22	26	29(2)	31	45	5
CGTA	26	57.62	55	56	58(6)	59	61	3
AGAG	26	46.77	36	42	48(3)	50	54	8
ATGC	22	48.91	33	48	51(4)	52	63	4
CACC	22	34.27	23	30	37(4)	38	40	8
AGGC	21	36.62	31	35	37(4)	38	41	3
TGCT	21	49.71	34	46	50(4)	51	75	5
TCCC	19	29.16	13	24	28(2)	31	54	7
GTGG	18	46.50	42	45	46(5)	47	52	2
GTTC	18	51.56	46	48	52(3)	53	64	5
CTCC	15	30.53	25	26	27(4)	28	53	2
GGTG	15	46.20	38	43	47(3)	48	52	5
AGTG	12	50.33	42	49	50(3)	52	60	3
GTGA	12	47.83	36	43	50(1)	52	57	9
GGCA	9	35.67	31	34	36(2)	37	39	3
GAGA	9	49.44	32	39	42(1)	47	82	8
CCCA	9	33.22	17	29	37(2)	39	43	10
CTAC	8	53.00	29	58	59(2)	61	65	3
CAGG	8	46.75	44	45	47(3)	49	50	4
GCAT	8	35.63	32	33	36(2)	38	39	5
ACAG	8	58.25	51	56	59(1)	62	66	6
AGAC	8	37.25	28	32	38(1)	39	48	7
CAGA	8	32.88	27	29	36(1)	37	38	8
TGTG	8	45.50	36	41	44(1)	50	57	9
GTGT	7	54.14	49	49	51(1)	59	61	10
CAGT	6	55.83	50	51	56(1)	61	62	10
TCAG	5	34.40	29	30	36(1)	38	39	8
GGAC	4	37.50	36	36	38(1)	40	40	4
GGCC	4	36.75	33	36	38(1)	40	40	4
GGTC	4	47.00	44	45	46(1)	53	53	8
ATATA	1479	62.12914131	1	60	63(123)	66	96	6

TATAT	1155	62.42597403	1	60	64(82)	68	96	8	
TGTAA	130	54.4	1	54	57(15)	59	91	5	
AATAC	129	57.65116279	2	56	61(5)	64	90	8	
ATACT	113	59.30973451	27	58	60(14)	62	91	4	
TATAC	100	61.2	46	60	62(12)	63	78	3	
ATACC	80	58.3125	20	58	60(13)	62	67	4	
CATTA	61	45.26229508	2	43	46(5)	49	89	6	Many peaks
CTCAT	57	46.33333333	11	46	48(7)	50	59	4	
GTATT	56	53.375	7	50	54(2)	59	95	9	
TACTG	53	58.83018868	53	57	59(11)	60	69	3	
ACTGT	53	57.88679245	51	56	58(12)	59	68	3	
ATGTA	51	57.03921569	3	55	59(7)	61	93	6	
CTGTA	49	56.7755102	41	55	57(10)	59	67	4	
GTAAT	47	57.21276596	18	52	56(3)	62	92	10	
TTCTC	46	47.7826087	29	43	49(5)	51	57	8	
GGGGG	44	48.79545455	42	46	49(5)	51	58	5	
TCTCA	43	49.18604651	38	43	48(5)	51	90	8	
TGTAT	41	59.31707317	42	54	58(4)	62	75	8	
ATTAC	38	57.60526316	5	56	61(5)	64	87	8	
TAATG	37	57.54054054	19	53	60(3)	62	95	9	
TAAGG	35	38.71428571	23	37	39(4)	41	60	4	
GAGGG	34	46.35294118	38	45	46(7)	48	54	3	
ACCAT	32	55.28125	23	57	58(10)	59	61	2	
TATAG	31	58.22580645	9	57	60(3)	62	92	5	
TACCT	31	55	19	54	58(3)	60	64	6	
CCATA	29	52.65517241	23	55	57(7)	58	60	3	
ATATG	29	59.27586207	17	55	59(2)	65	95	10	
TACCA	28	58.35714286	44	58	59(8)	60	62	2	
AGGGT	27	45.62962963	42	44	45(4)	47	50	3	
TACCG	24	59.95833333	57	58	60(7)	61	64	3	
AGGGG	24	48.25	38	47	49(5)	50	55	3	
ATCCT	24	51.5	28	50	52(3)	54	71	4	
TCCTC	23	50.39130435	47	48	50(2)	52	58	4	
ACCGT	22	59.13636364	56	58	59(7)	60	63	2	
ACCTT	22	58	51	57	59(5)	60	61	3	
CCTCA	22	47.77272727	26	47	50(4)	51	57	4	
AATCC	22	49.45454545	17	49	51(3)	53	64	4	
GGAGG	22	47.18181818	43	45	47(6)	49	52	4	
GGGTA	21	44.66666667	40	43	44(3)	46	51	3	
TAGGG	20	47.05	44	45	47(2)	49	52	4	
AGGGA	19	45.47368421	37	44	46(4)	47	51	3	
AAGGG	19	49	44	46	49(3)	51	57	5	
ATAGT	19	60.68421053	25	56	59(2)	63	92	7	
CCAAA	18	23.27777778	5	19	23(2)	25	39	6	
ACTAT	17	53.58823529	17	54	58(2)	59	76	5	
ATTGT	17	56.76470588	10	56	59(3)	61	87	5	

AAGGA	17	45.58823529	35	39	45(1)	49	59	10
CCGTA	15	58	56	57	58(5)	59	61	2
TTACC	15	56.13333333	26	59	60(3)	62	63	3
ACCCT	14	33.14285714	25	29	30(1)	31	55	2
GGTAT	14	45.92857143	39	43	44(5)	45	64	2
TTCCT	14	55.57142857	47	49	50(2)	53	88	4
CCCTC	13	28.15384615	25	27	28(3)	29	31	2
AGAGG	13	47.69230769	40	47	49(2)	50	51	3
TACTC	13	46.92307692	1	49	52(1)	58	63	9
GGTAG	12	46.91666667	42	45	46(3)	47	57	2
AAGGC	12	37.08333333	33	36	38(3)	39	41	3
AAAGG	12	53.58333333	47	51	55(1)	57	58	6
ACTCT	12	53.41666667	37	50	54(1)	60	62	10
GGGTT	11	44.18181818	41	43	44(1)	45	48	2
GGGGA	11	45.45454545	37	43	46(1)	47	52	4
TCCTT	11	44.36363636	18	46	47(1)	50	56	4
GGGAT	11	44.36363636	36	42	45(2)	47	49	5
CTCTA	11	49.18181818	31	45	51(2)	53	61	8
GTGGG	10	46.1	44	45	46(2)	47	51	2
GGTAA	10	41.6	21	39	41(3)	43	59	4
AGGTA	10	49.3	42	44	49(1)	50	59	6
ATAGG	10	50.7	44	45	53(2)	54	57	9
GGGAA	9	43.88888889	38	42	43(2)	44	55	2
GGGAG	9	47.66666667	45	46	48(2)	49	51	3
AAGAG	9	47.77777778	36	47	50(2)	52	54	5
CCCTT	9	28.77777778	23	25	30(2)	31	35	6
TAGGT	9	43.44444444	9	45	51(1)	55	59	10
AGGTT	9	39.33333333	5	39	48(1)	49	56	10
TGGGG	8	48.75	45	48	49(2)	50	55	2
TACCC	8	31	26	30	32(3)	33	34	3
CTCAC	8	37.25	25	37	40(2)	41	50	4
CCCCT	8	27.875	22	26	29(1)	30	34	4
CAGGG	8	46.75	44	45	47(3)	49	50	4
CGTAA	7	57.42857143	55	56	57(2)	58	60	2
ACCCC	7	33.14285714	26	29	31(2)	32	45	3
GGGTG	7	44.71428571	38	43	44(1)	47	50	4
GGTTA	7	43	38	40	42(2)	45	48	5
TAAGC	7	48.71428571	31	36	39(1)	41	80	5
CACCC	7	30.85714286	23	26	30(2)	32	39	6
TCACC	7	35.57142857	29	31	37(1)	38	40	7
AACAC	7	45.42857143	28	38	41(1)	45	67	7
TGGGT	6	45.33333333	44	44	45(2)	46	48	2
CCCCC	6	26.33333333	24	25	26(1)	27	31	2
GGTGG	6	47.33333333	42	46	48(1)	49	52	3
GAGGA	6	46.5	44	44	47(2)	48	49	4
GTTCT	6	53	47	49	52(2)	54	64	5

GTAGG	6	48.33333333	45	45	46(2)	50	58	5
AGGAA	6	47.66666667	39	45	48(1)	52	55	7
TCTCT	5	49.6	45	48	51(1)	52	52	4
ACCCA	5	36.4	29	35	37(1)	39	42	4
TCCCC	5	28.8	24	26	28(1)	30	36	4
CTCCC	5	28.4	25	26	28(1)	31	32	5
TACAG	5	59.4	52	57	59(1)	63	66	6
TGAAG	5	26.4	16	23	24(1)	31	38	8
TTTCC	5	52.6	48	48	55(1)	56	56	8
CAGAA	5	32	27	28	30(1)	37	38	9
TGTAG	5	55.8	50	50	59(1)	60	60	10
AGTTG	5	44	32	40	44(1)	50	54	10
CACCA	4	37	34	37	38(1)	39	39	2
TTAGG	4	11.75	10	11	12(1)	14	14	3
AGGCC	4	36.75	33	36	38(1)	40	40	4
AGGAC	4	37.5	36	36	38(1)	40	40	4
ACTAC	4	61.75	59	59	64(1)	65	65	6
GCATT	4	35.25	32	32	38(1)	39	39	7
TCACT	4	46	40	44	49(1)	51	51	7
TACAC	4	54.25	46	53	57(1)	61	61	8
CCTAA	4	27	20	24	30(1)	34	34	10
AAAGC	4	58.75	55	56	58(1)	66	66	10
CCTAT	4	51.25	44	49	53(1)	59	59	10
AGACC	4	37.75	28	37	39(1)	47	47	10
TGTTC	3	49	48	48	49(1)	50	50	2
GTGAT	3	49.33333333	48	48	49(1)	51	51	3
TGGGA	3	44.33333333	43	43	44(1)	46	46	3
TAACC	3	33.66666667	32	32	34(1)	35	35	3
CATAC	3	59.66666667	58	58	59(1)	62	62	4
AGTGA	3	52	50	50	52(1)	54	54	4
AGTAG	3	48.33333333	46	46	48(1)	51	51	5
CCTTC	3	27.66666667	24	24	29(1)	30	30	6
GTTCA	3	48.66666667	46	46	48(1)	52	52	6
TTCCA	3	8.666666667	5	5	9(1)	12	12	7
GGTTC	3	51.66666667	47	47	53(1)	55	55	8
GTTTG	3	43	39	39	43(1)	47	47	8
TAGCA	3	36.33333333	32	32	36(1)	41	41	9
CACAA	3	7.666666667	4	4	6(1)	13	13	9
TCAGA	3	34.33333333	29	29	36(1)	38	38	9
GGAAG	3	50.33333333	45	45	51(1)	55	55	10
AACCC	3	34	30	30	32(1)	40	40	10
CCCTA	3	26	21	21	26(1)	31	31	10
GTAGA	3	48.66666667	45	45	46(1)	55	55	10

*Three IRs counted less than 3 were removed from the list.

Table S5 IES positions among strains

*Table S5 is in a separated excel file.

Table S6. Distributions of flanking G and C motifs in LIA3-affected IESs in CU427.

			**LIA3-affected candidate IESs**			**LIA3-unaffected IESs**		
Constrain[1]	IES no.	Similarity[2]	G IR[3]	C IR[4]	%	G IR[3]	C IR[4]	%[5]
WT ≤ 10 bp (WT-LIA3Δ) ≥ 100 bp	230	60%	159	62	96.09	1598	2194	73.87
		75%	120	41	70	350	421	15.02
WT ≤ 20 bp (WT-LIA3Δ) ≥ 100 bp	308	60%	208	83	94.48	1549	2173	73.63
		75%	158	52	68.18	312	410	14.28
		80%	117	35	49.35	162	145	6.07
WT ≤ 30 bp (WT-LIA3Δ) ≥ 100 bp	326	60%	217	89	93.87	1540	2167	73.60
		75%	164	56	67.48	306	406	14.14
WT ≤ 100 (WT-LIA3Δ) ≥ 100 bp	387	60%	247	106	91.21	1519	2150	73.73
		75%	175	62	61.24	295	400	14.02

[1]The difference between IESs among three inbred strains is less than or equal to the indicated number of base pairs (bp), and the IES differences between the three inbred and three LIA3Δ strains are ≥ 100 bps.

[2]The similarity of PWM score of the consensus indicated in Fig. 5A and 5B.

[3]Number of IES candidates containing G-rich IRs.

[4]Number of IES candidates containing C-rich IRs.

[5]Percentage of IESs in the WT background without candidates of G-rich and C-rich IRs.

*The numbers of IRs are non-overlapped between each group. If G-rich and C-rich IRs appeared in the same IES, the one that has less distance difference to IES boundary between each copy was selected as the FRS for the IES.

Table S7 Average distance of LIA3-affected IESs with G-rich or C-rich IRs (WT ≤ 100 bp and (WT-LIA3Δ) ≥ 100 bp) in CU427.

Similarity: 60%	**(bp)**	**A-distance**	**B-distance**	**PWM**	**Distance difference**
**LIA3-affected IESs with G-rich IR**	Average	51.60	50.98	2.95	3.03
	s.d.	8.47	10.26	0.45	6.92
**LIA3-affected IESs with C-rich IR**	Average	40.36	41.46	2.84	5.86
	s.d.	18.27	20.58	0.40	11.36

Table S8 Class interval of G-rich IRs and C-rich IRs in LIA3-affected IESs.

Maximum variation in WT strains (bp)	Count	
	G IR	C-IR
0	43	16
1-10	77	25
11-20	38	12
21-30	6	3
31-40	0	2
41-50	2	2
51-60	3	0
61-70	3	2
71-80	2	0
81-90	0	0
91-100	1	0
Total	175	62

Table S9 The position of the T-domain containing IES,

CU427.Supercontig2.222.9221

strain	Pos1	Pos2	length
BII-1	57,625	67,745	10,121
BII-2	68,653	78,805	10,153
CU427	57,620	78,805	21,186
CU428-1	57,625	67,745	10,121
CU428-2	68,653	78,805	10,147

Table S10 LIA3-affected IESs with C-rich IRs

Table S11 LIA3-affected IESs with G-rich IRs

*Table S10 and S11 are in separated excel files.

Table S12 Primer information

Test the rearrangement of the IES with the C-rich IR	
FOR1	catagtagttgcttgagcttttgaag
REV1	gatccgggcaacgttgttgc
REV2	cgcttgaaattaaagtgggataaag
Test the rearrangement of the IES with the T-domain	
FOR2	ctgctggggttgttaactcttaag
REV3	gagaaaggctctgggactgc
